# Continuous-wave Doppler interrogation in valvular heart disease: pearls and pitfalls

**DOI:** 10.1093/ehjimp/qyag096

**Published:** 2026-05-21

**Authors:** Frank Timmermans, Edgar Argulian, Hatem Soliman-Aboumarie

**Affiliations:** Department of Cardiology, University Hospital Ghent, Ghent University, Corneel Heymanslaan 11, Ghent 9000, Belgium; Department of Cardiology, Mount Sinai Morningside, Icahn School of Medicine at Mount Sinai, 1111 Amsterdam Avenue, NewYork, NY 10025, USA; Department of Cardiac Anaesthesia and Critical Care, Harefield Hospital, Royal Brompton and Harefield Hospitals Hill End Road, Uxbridge UB9 6JH, UK; School of Cardiovascular, Metabolic Medicine and Sciences, King’s College London, Strand, London WC2R 2LS, UK

**Keywords:** continuous-wave Doppler, echocardiography, valvular heart disease, Doppler haemodynamics

## Abstract

Continuous-wave Doppler (CWD) echocardiography remains a cornerstone in the haemodynamic assessment of valvular heart disease, providing critical insights into transvalvular flow, pressure gradients, and lesion severity. Beyond simple velocity measurements, the morphology, timing, intensity, and contour of Doppler waveforms contain rich physiological information that can refine diagnosis, risk stratification, and clinical decision-making. However, these signals are frequently underutilized or misinterpreted because accurate interpretation requires meticulous acquisition technique, understanding of Doppler physics, and integration with haemodynamic principles. This review discusses the fundamental principles underpinning CWD interrogation, including the Doppler and Bernoulli equations, and highlights the strengths, limitations, and common pitfalls encountered in clinical practice. We systematically review the application of CWD in aortic, mitral, tricuspid, and pulmonary valve disease, focusing on waveform morphology, velocity and gradient interpretation, pressure half-time, velocity–time integral, dimensionless indices, and Doppler signal intensity analysis. Particular emphasis is placed on complex haemodynamic scenarios, including low-flow states, dynamic obstruction, mixed valve disease, severe regurgitation, prosthetic valves, and discordant findings between Doppler and invasive measurements. The review also explores emerging concepts such as Doppler signal pixel intensity analysis and the future role of artificial intelligence in integrating high-density Doppler information into physiology-driven interpretation frameworks. Ultimately, Doppler echocardiography should not be viewed merely as a source of numerical outputs, but as a comprehensive bedside physiological tool requiring critical interpretation within the broader clinical and haemodynamic context.

## Graphical Abstract

**Graphical Abstract:**
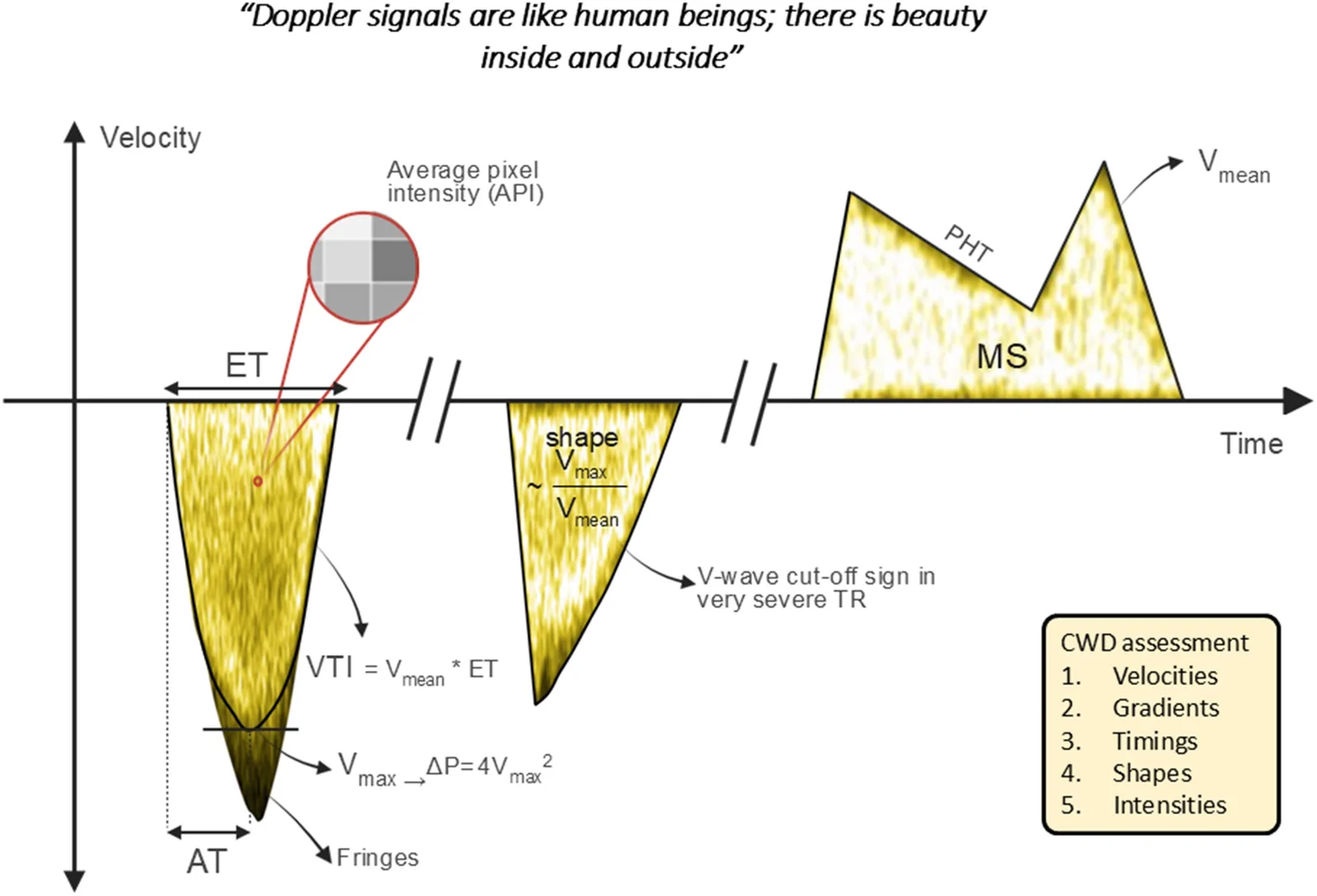

## Introduction

The evolution of echocardiography has profoundly transformed the evaluation and management of valvular heart disease, enabling refined diagnostic accuracy and improved risk stratification. Among the echocardiographic modalities, Doppler interrogation provides an extensive array of haemodynamic data reflecting the severity and pathophysiologic consequences of valvular lesions. The contour of the Doppler spectrum, its peak and mean velocities, acceleration and deceleration slopes, signal intensity, timing characteristics, and integrative indices all carry important diagnostic and prognostic information. However, this wealth of information is often underrecognized, underutilized, or subject to misinterpretation. Accurate assessment and interpretation of Doppler signals require meticulous adherence to technical acquisition principles and a thorough understanding of underlying haemodynamic concepts.

The current review provides an overview of the strengths, limitations, and interpretative considerations of Doppler assessment in valvular heart disease, largely focusing on continuous-wave Doppler (CWD).

### From Doppler to velocity: the Doppler equation

**Figure 1:**
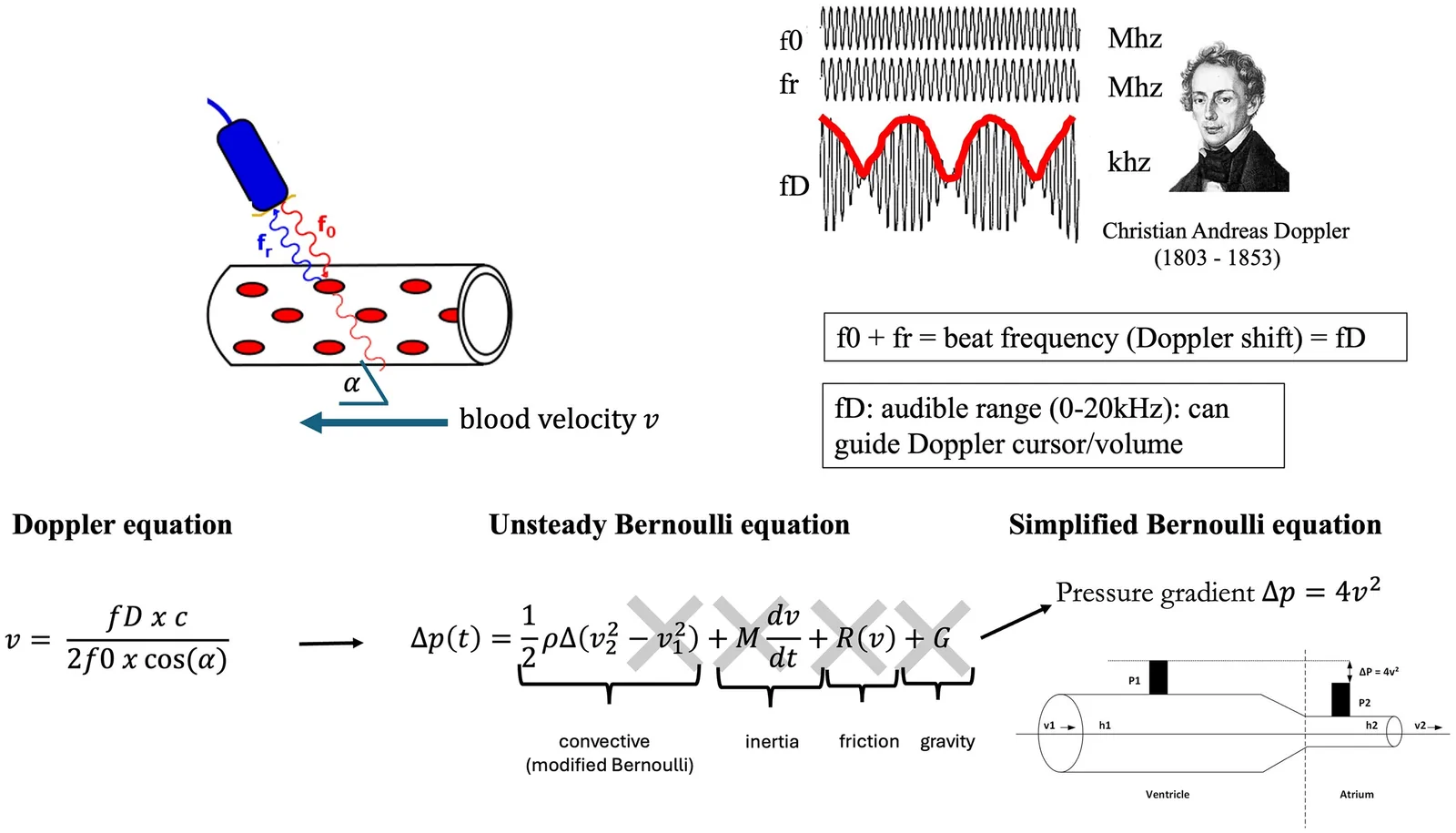
From Doppler velocity towards pressure gradients. Transmission (f0) and reception (fr) of ultrasound waves are ‘summed’, yielding the Doppler-shifted frequencies (fD), allowing the calculation of velocities using the Doppler equation (bottom left). The velocity *v* is affected by the angle between orientation of blood flow and Doppler beam. The pressure gradient Δp is mainly determined by the convective flow component, therefore omitting the other terms (see text), eventually yielding the modified Bernoulli equation, i.e. $\Delta p=4\;(v_{2}^{2}-\;v_{1}^{2})$. Omitting $v_{1}\;$ yields the simplified Bernoulli equation which reflects the static pressure difference between P1 and P2, as illustrated by the narrowing tube model (bottom right).

**Figure 2:**
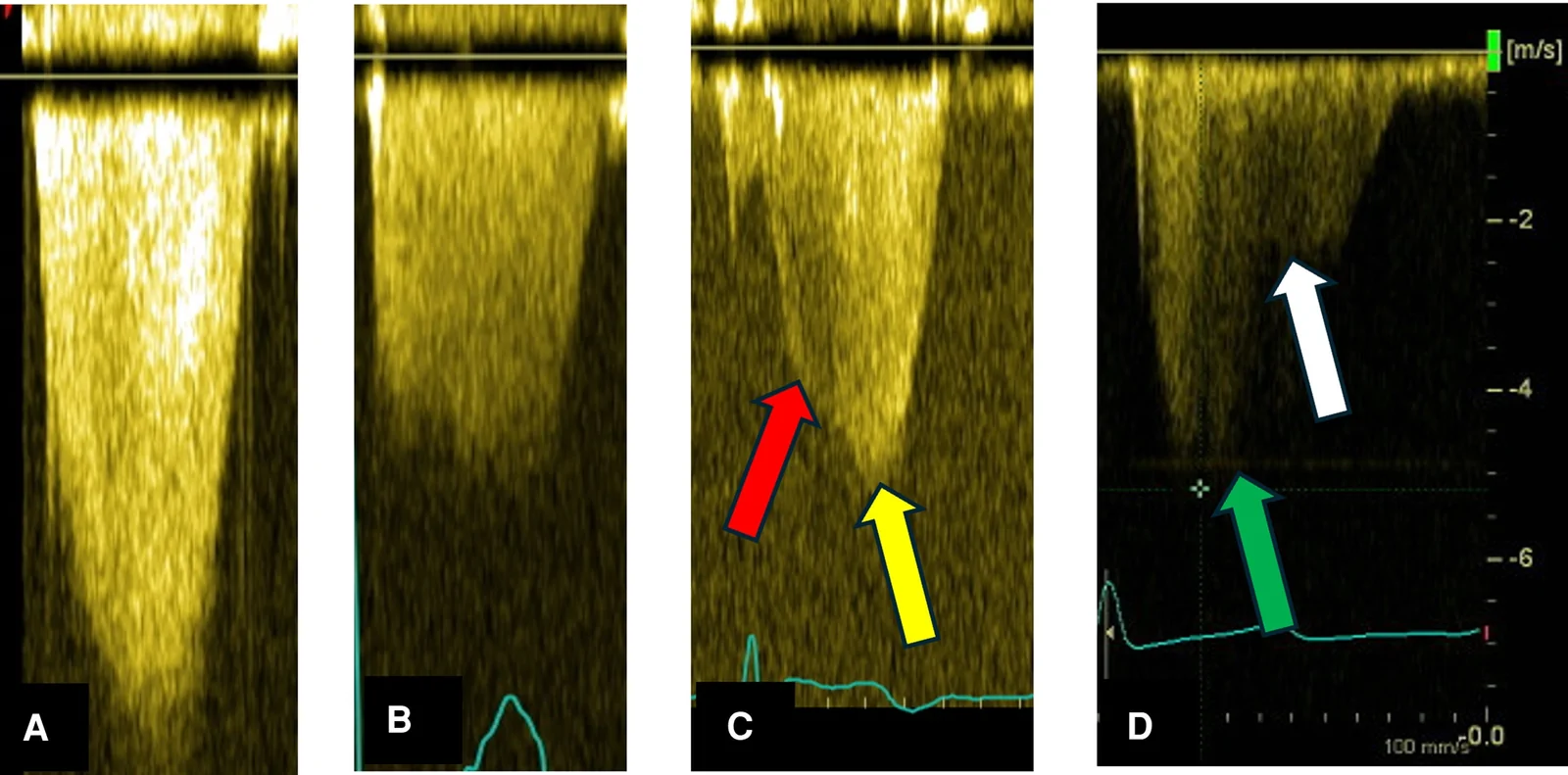
Pitfalls of spectral Doppler interrogation. (*A*) shows a well-aligned, blunted tricuspid regurgitation (TR) CWD signal in a patient with pulmonary arterial hypertension (right ventricular systolic pressure of 65 mmHg), whereas (*B*) shows a truncated TR CWD signal due to misalignment of the beam relative to the TR jet in the same patient; (*C*) shows a double TR CWD signal, due to mild prolapse of the tricuspid valve, creating two distinct parts of the CWD Doppler signal (red and yellow arrow); (*D*) shows a double ‘TR’ signal, which is due to contamination from an apical ventricular septal defect signal with left-to-right shunt (green arrow), creating a falsely high right ventricular pressure gradient of 107 mmHg. The white arrow indicates the TR CWD signal.

Continuous-wave Doppler uses the Doppler principle to derive blood velocities. The Doppler principle is based on the frequency shift (Doppler shift) that occurs when ultrasound waves are reflected by moving red blood cells (RBCs). The Doppler-shifted frequency represents the difference between the transmitted frequency and the received frequency (i.e. the beat frequency) allowing the calculation of blood flow velocity using the Doppler equation (*Figure 1*). Because ultrasound waves are emitted continuously and the reflected signals are sampled without interruption, CWD does not experience aliasing. This contrasts with pulsed wave Doppler (PWD), which has a finite sampling rate determined by the pulse repetition frequency (PRF): aliasing with PWD results from undersampling of the signal and leads to ‘wraparound’ of the spectral Doppler tracing appearing as the flow in the opposite direction. In CWD, the continuous transmission and reception of ultrasound waves enable accurate measurement of high velocities but preclude precise spatial localization of flow along the Doppler beam.^1–3^ In clinical practice, accurate interpretation therefore relies on visual inspection of the spectral display, anatomical knowledge, understanding of flow regions, and the characteristic pitch and contour or shape of the Doppler signal (*Figure 2*). The Doppler equation assumes perfect alignment between the ultrasound beam and the direction of blood flow (i.e. the cosine of the insonation angle equals one) (*Figure 1*). Therefore, minimizing the angle between the CWD beam and the flow jet is essential to ensure accurate velocity estimation.^2^

The outer edge of the CWD signal reflects the maximal velocities captured by the interrogating beam at any time point and corresponds to the flow at the narrowest zone (the vena contracta) along the beam. At any time point of the outer edge of the CWD signal, the recorded peak velocity is driven by the instantaneous pressure gradient, and this pressure gradient changes in time, hence shaping the Doppler signal. A truncated CWD spectral envelope typically indicates misalignment of the Doppler beam with the vena contracta or flow jet, resulting in underestimation of velocities. However, apparent truncation can also occur due to haemodynamic phenomena, such as in cases of massive or torrential tricuspid regurgitation (TR).^4^ Incomplete CWD signals may also be attributed to translational movements of the jet relative to the CWD beam or in cases of dynamic/mild regurgitation with protosystolic or telesystolic signals or midsystolic dipping of regurgitation causing midsystolic signal void of the CWD signal (see below). Furthermore, as the interrogating area of the CWD is not small (it mostly spans < 1 cm^2^ at the focus point and is determined by physical properties such as ultrasound frequency, probe geometry, and depth of interrogation^5^), the CWD beam may capture signals outside the 2D imaging plane, especially within the elevation plane, causing contamination from remote flow signals not captured in the 2D image (beam width effect).^6^ Furthermore, to minimize alignment errors in CWD assessment, it is essential to (i) obtain multiple acquisitions from different imaging windows and insonation angles [e.g. use of a dedicated non-imaging transducer at the right parasternal window for aortic stenosis (AS)]; (ii) utilize auditory feedback by listening to the Doppler signal, as the pitch provides additional clues regarding optimal alignment;^2^ and (iii) use of colour Doppler in regurgitant lesions to align the regurgitant jet with the CWD beam.

**Figure 3:**
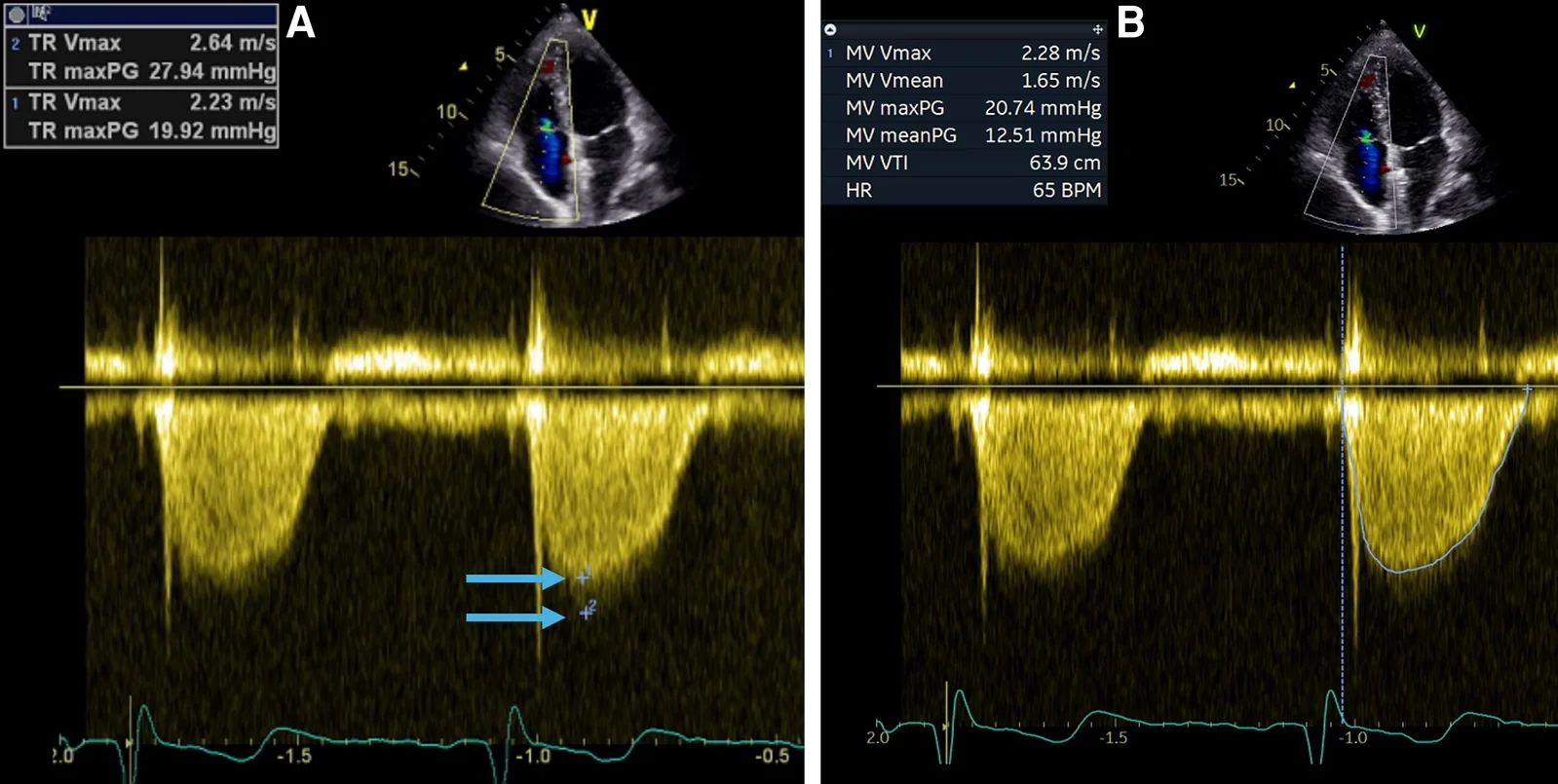
Assessment of Doppler velocities at the outer modal velocity spectrum. TR velocity at the top of the fringes shows a Vmax of 2.64 m/sec, whereas a significant lower Vmax of 2.23 m/sec is obtained when measuring at the outer edge of the intense modal velocity (*A*). Tracing of the TR signal to obtain the VTI also occurs at the outer edge of the modal velocity, thus excluding the fringes (*B*).

Overestimation of Doppler velocities may also occur, most commonly due to inaccurate caliper placement during measurement, assuming correct Doppler envelope is identified. To avoid this error, peak velocity should be traced along the outer edge of the most intense portion of the signal, the so-called modal velocity, while excluding fringes and faint linear artefacts, the latter caused by the transit-time effect. Transit-time effect refers to spectral broadening and blurring that occurs due to the short transit time of RBCs within the limited width of the Doppler beam resulting in a broad frequency spectrum and hence uncertainty in velocity estimation.^7^ As a practical rule, Doppler contour measurements should be made ‘at the chin, not at the beard’.^3^ The same applies when tracing the velocity–time integral (VTI) (*Figure 3*).^2^ Artificial intelligence (AI) algorithms do not necessarily exclude these Doppler fringes, leading to overestimated peak velocities and gradients, and although some vendors allow to adjust thresholds for automatic detection, visual inspection of the caliper positioning remains mandatory.

Apart from suboptimal (dynamic) misalignment of the CWD beam relative to the regurgitant or stenotic jet, the shape of the CWD signal is fundamentally governed by the components of Ohm’s law, i.e. flow (rate), pressure gradient and impedance, and dynamic interaction between these components during the flow event. Specific shapes harbour relevant information on the haemodynamic severity of the valve lesion and will be discussed separately for each valve lesion.

Finally, accurate Doppler measurements are dependent on proper machine settings, particularly gain and wall filter adjustments. Careful adjustment of gain and wall filter is essential to obtain a clear, well-defined Doppler envelope for accurate tracing and velocity assessment.^8^ In practice, the CWD gain should be gradually increased until the spectral signal is well visualized and then slightly reduced to eliminate background noise and avoid overfilling of the envelope. Excessive gain can exaggerate spectral broadening, leading to overestimation of velocities, whereas insufficient gain may obscure low-amplitude components of the Doppler signal and result in underestimation.^8^ The wall filter is an important Doppler machine setting that eliminates low-frequency, high-amplitude signals caused by slow-moving structures such as heart walls and valves; the wall filter should be set low enough to preserve low-velocity components of flow, but high enough to suppress baseline noise and motion artefacts. Optimal settings result in a crisp outer envelope with minimal spectral clutter, allowing precise delineation of peak velocity and contour tracing. If ultrasonic enhancing agents are used to enhance Doppler recordings, significant blooming of the Doppler signal is observed creating a great potential for overmeasuring the envelope. Careful adjustment of gain is necessary to only display the intense, modal contour of the Doppler envelope.^9,10^ In *Table 1*, a summary is provided on pitfalls in CWD assessment.

**Table 1:** Summary of pitfalls in continuous-wave Doppler velocity assessment

Pitfall	Underlying mechanism	Impact on measurement	Practical clues/mitigation
Angle misalignment	Doppler equation assumes parallel alignment between ultrasound beam and flow (cos θ = 1)	Systematic underestimation of velocity	Use multiple windows; minimize insonation angle; rely on highest velocity signal
Lack of spatial localization (CWD)	Continuous transmission prevents depth resolution along the beam	Velocity may originate from unintended flow regions	Anatomical knowledge; integrate with 2D imaging and jet direction
Truncated spectral envelope	Beam does not intersect vena contracta or peak jet velocity	Underestimation of peak velocity	Adjust probe position; seek full, dense envelope
Physiological pseudo-truncation	Extreme regurgitation (e.g. massive/torrential TR) or rapid pressure equalization	Apparent truncation without misalignment	Interpret in clinical and haemodynamic context
Dynamic or intermittent regurgitation	Protosystolic, telesystolic, or midsystolic dipping of flow	Incomplete or interrupted Doppler envelope	Correlate with timing on ECG, 2D image and colour Doppler
Jet translation during systole	Movement of jet relative to fixed CWD beam	Signal dropout or incomplete envelope	Reacquire from alternative windows
Beam width and elevation–plane contamination	CWD samples outside imaging plane (<1 cm2 focal region)	Overlapping or remote flow contamination	Use multiple views; confirm signal origin
Incorrect envelope identification	Tracing faint fringes or artefacts rather than modal velocity	Overestimation of peak velocity and VTI	Trace the dense outer contour (‘at the chin, not the beard’)
Transit-time artefacts	Linear fringes adjacent to true signal	Artificial velocity exaggeration	Ignore faint peripheral lines
Inappropriate gain (too high)	Excessive amplification	Spectral broadening → overestimation	Reduce gain until modal contour is clear
Inappropriate gain (too low)	Poor signal amplification	Loss of low-amplitude components → underestimation	Increase gain carefully to visualize full envelope
Incorrect wall-filter settings	Removal of low-frequency signals	Inaccurate VTI, especially in low-velocity flows	Lower wall filter when assessing slow flows
Ultrasound enhancing agent (UEA) use	Signal blooming due to contrast	High risk of overmeasuring envelope	Reduce gain; trace only intense modal contour
Overreliance on single window	Limited insonation perspective	Missed true peak velocity	Systematic multiwindow interrogation
Ignoring auditory feedback	Visual data alone may be misleading	Suboptimal alignment	Use pitch intensity to optimize beam alignment

In summary, CWD enables accurate measurement of high blood flow velocities without aliasing by continuously transmitting and receiving ultrasound signals, but it lacks spatial resolution and therefore requires meticulous alignment and careful interpretation of the spectral envelope. Accurate assessment depends on integration of technical factors, including beam alignment and machine settings, with haemodynamic understanding of signal shape, intensity, and contour to avoid common sources of error.

### From blood velocities towards pressure gradients: Bernoulli rules

The Bernoulli principle describes the reciprocal relation between the velocity of a fluid in a tube and its static pressure measured at the tube’s surface (*Figure 1*). Understanding Bernoulli’s principle requires an energy-based rather than a purely pressure-based perspective. Bernoulli’s principle describes the conservation of energy in an incompressible, nonviscous fluid moving along a single streamline: each unit of blood flow contains kinetic energy (∼ dynamic pressure), potential energy (∼static pressure), and a small gravitational component, and the sum of these three components remains constant along the streamline, i.e. the total energy remains constant before, at, and downstream of the stenosis. Therefore, as blood accelerates through a stenotic orifice, kinetic energy increases (dynamic pressure increases), while potential energy decreases (static pressure decreases). Thus, Doppler echocardiography, by measuring changes in blood velocity, provides an indirect estimate of intracardiac pressure gradients.

The full unsteady Bernoulli equation includes convective, inertial, viscous, and gravitational components as shown in *Figure 1*.^11,12^ In clinical Doppler applications, only the convective term is retained, as the other components contribute minimally to pressure differences across cardiac valves (*Figure 1*).^11^ This simplification yields the modified Bernoulli (MB) equation, which forms the basis of Doppler pressure (gradient) estimation. In most clinical settings, however, the proximal velocity (v_1_) is eventually neglected when its magnitude is small (<1.5 m/s) or when it is much lower than the distal velocity (v_2_), leading to the familiar simplified Bernoulli (SB) equation (ΔP = 4v^2^) (*Figure 1*).^1,11^ This simplification accurately describes flow through stenotic or regurgitant orifices under physiologic conditions. In fact, the SB equation remains the cornerstone of Doppler haemodynamics and has largely supplanted invasive pressure measurements in the evaluation of valvular stenosis and regurgitation. Invasive haemodynamic assessment measures pressure differences between two points, representing the aggregate of all pressure changes across the measured segment. Because pressure has no directional component, invasive gradients reflect the integrated pressure profile rather than segmental variations. In contrast, CWD interrogation captures localized instantaneous pressure gradients, offering superior temporal resolution but with pitfalls under certain haemodynamic conditions. However, invasive haemodynamic assessment remains valuable in selected clinical scenarios, particularly in the presence of discordant echocardiographic and clinical findings or complex haemodynamic derangements.^13,14^

In summary, Bernoulli’s principle underpins Doppler haemodynamics by linking increases in flow velocity to reciprocal decreases in static pressure, allowing pressure gradients to be inferred from velocity measurements. In clinical practice, this relationship is simplified using adapted Bernoulli equations, enabling Doppler echocardiography to provide accurate, noninvasive estimation of transvalvular pressure gradients, albeit with important physiologic and technical limitations.

In the following section, for each valve disease, we will systematically discuss CWD velocities and gradients, the shape of the CWD waveform, specific time-interval measures and indices, and other, more valve-specific measures such as VTI, the dimensionless index (DI), pressure half-time (PHT), and CWD signal pixel intensity.

## Aortic valve disease

### Aortic stenosis

**Figure 4:**
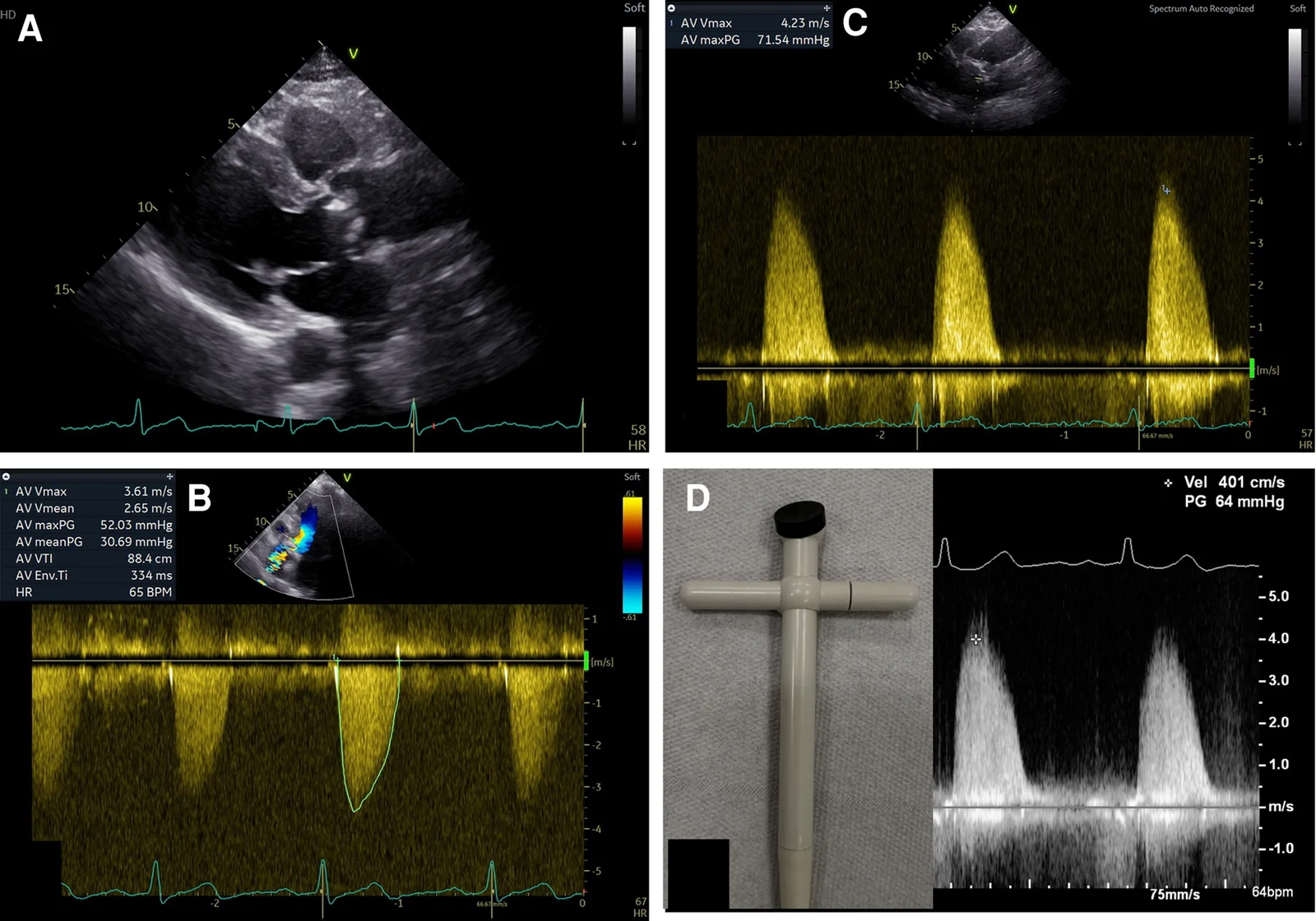
Impact of insonation angle and pressure gradients in aortic stenosis. (*A*) shows a highly calcified bicuspid aortic valve with a Δp max. of 52 mmHg obtained in the apical chamber view (*B*). A modified left parasternal long axis view shows a Δp max. of 71.5 mmHg (*C*), whereas pencil probe interrogation at the right parasternal view yields a Δp max. of 64 mmHg (*D*).

**Figure 5:**
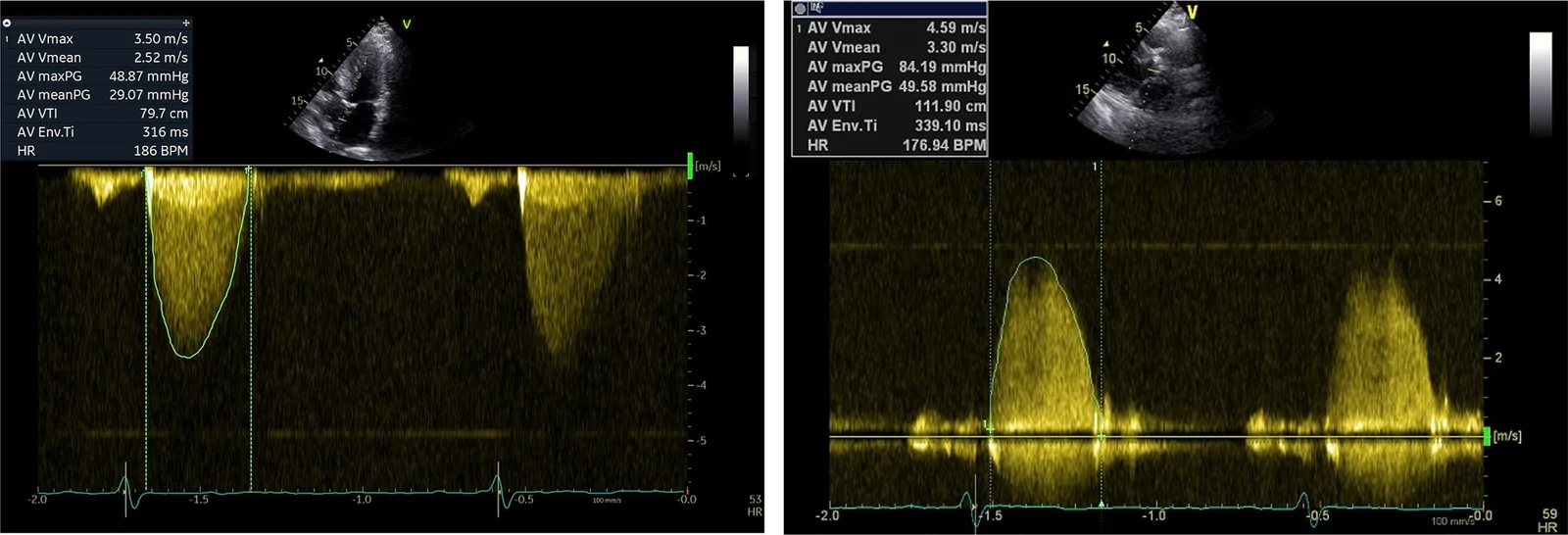
From apparent moderate towards severe aortic stenosis. In the AP5CH view, CWD interrogation yields a Pmean of 29 mmHg with an AS–VTI of 79.9 cm and an AVA of 1.2 cm^2^ suggesting moderate AS (left panel); in the modified parasternal long axis view with conventional 2D probe (right panel), a Pmean of 49 mmHg is obtained with an AS–VTI of 111.9 cm, yielding an AVA of 0.79 cm^2^, consistent with high-gradient severe AS.

Velocities^15^ and transvalvular pressure gradient^16^ measurements are integral components of AS severity assessment. Accurate CWD alignment is essential, as beam–flow angles exceeding 20° can result in substantial underestimation of peak velocity, whereas smaller deviations (<20°) typically introduce errors of < 10%. In one study, the highest velocity was located outside the apical imaging window in 61% of cases; neglecting non-apical windows led to misclassification of stenosis severity in 23% of patients (*Figure 4*).^17^ Thus, discordance between pressure gradients and calculated aortic valve area (AVA), such as encountered in (i) in low-gradient (Pmean < 40 mmHg) small AVA (AVA < 1.0 cm^2^), (ii) high-gradient AS [mean gradient (Pmean) > 40 mmHg] with large AVA (AVA > 1 cm^2^) referred to as ‘pseudo-moderate AS’, and (iii) even apparent moderate AS (Pmean < 40 mmHg and AVA >1.0 cm^2^), can be simply explained by suboptimal Doppler velocity assessments in a substantial number of patients. In *Figure 5*, optimal Doppler assessment from a modified parasternal long axis reclassified moderate AS into a high-gradient severe AS, as higher CWD velocities captured in this modified window also significantly increased the VTI and, hence, resulted in a smaller calculated AVA. In addition to suboptimal Doppler velocity assessments, the frequently encountered discordant finding of AVA < 1 cm^2^ and Pmean < 40 mmHg can also be attributed to (i) suboptimal left ventricular outflow tract (LVOT) area calculation, (ii) low-flow rate states, (iii) indexing AVA by body surface area, (iv) inherent hydraulic inconsistencies in guideline cut-off criteria between AVA and Pmean and, (v) pseudo-normalization of AS gradients in patients with arterial hypertension and/or reduced arterial compliance.^18–21^

By virtue of its time-integrative nature, Pmean closely correlates with invasively measured Pmean gradients in native AS, and it represents a clinically validated parameter for grading lesion severity and prognostication.^16^ In contrast to the peak gradient (Pmax), Pmean integrates the entire systolic flow profile and is therefore more reproducible and less sensitive to signal noise. In this regard, transit-time linear artefacts and especially the fringes at the peak of the CWD signal in AS should be excluded when measuring peak Doppler velocities and gradients.^22^ These fringes are due to the fact that the velocity streamline profile in (severe) AS is not flat and the SB equation assumes a single streamline principle, neglecting the 3D flow profile through a stenotic orifice.^12^ Theoretically and physiologically, contrary to Pmax, Pmean in severe AS is expected to better correlate with outcomes as Pmean is a surrogate for prolonged contraction in severe AS.^23^ Yet, both Pmax and Pmean gradients have been similarly validated for clinical assessment and risk stratification.^16^

**Figure 6:**
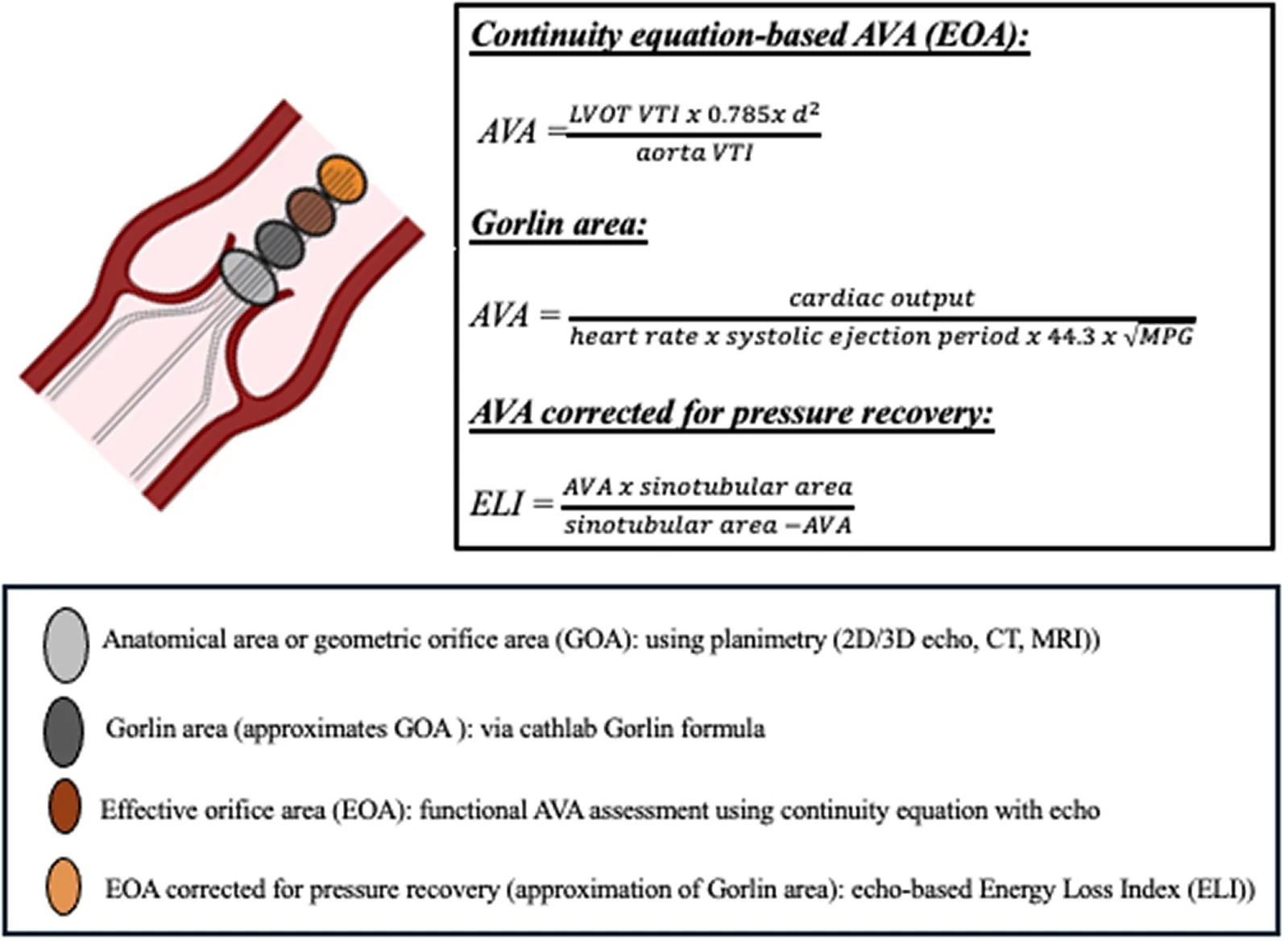
Aortic valve area nomenclature. The anatomical AVA is assessed by direct planimetry using computed tomography, cardiac magnetic resonance, or 2D/3D echocardiography, whereas the Gorlin-based AVA used for invasive assessment of AS severity is an approximation of the anatomical AVA. Doppler echocardiography using the continuity equation assesses the effective orifice area (functional AVA in case of AS). The ELI is the AVA corrected for pressure recovery.^24,25^

**Figure 7:**
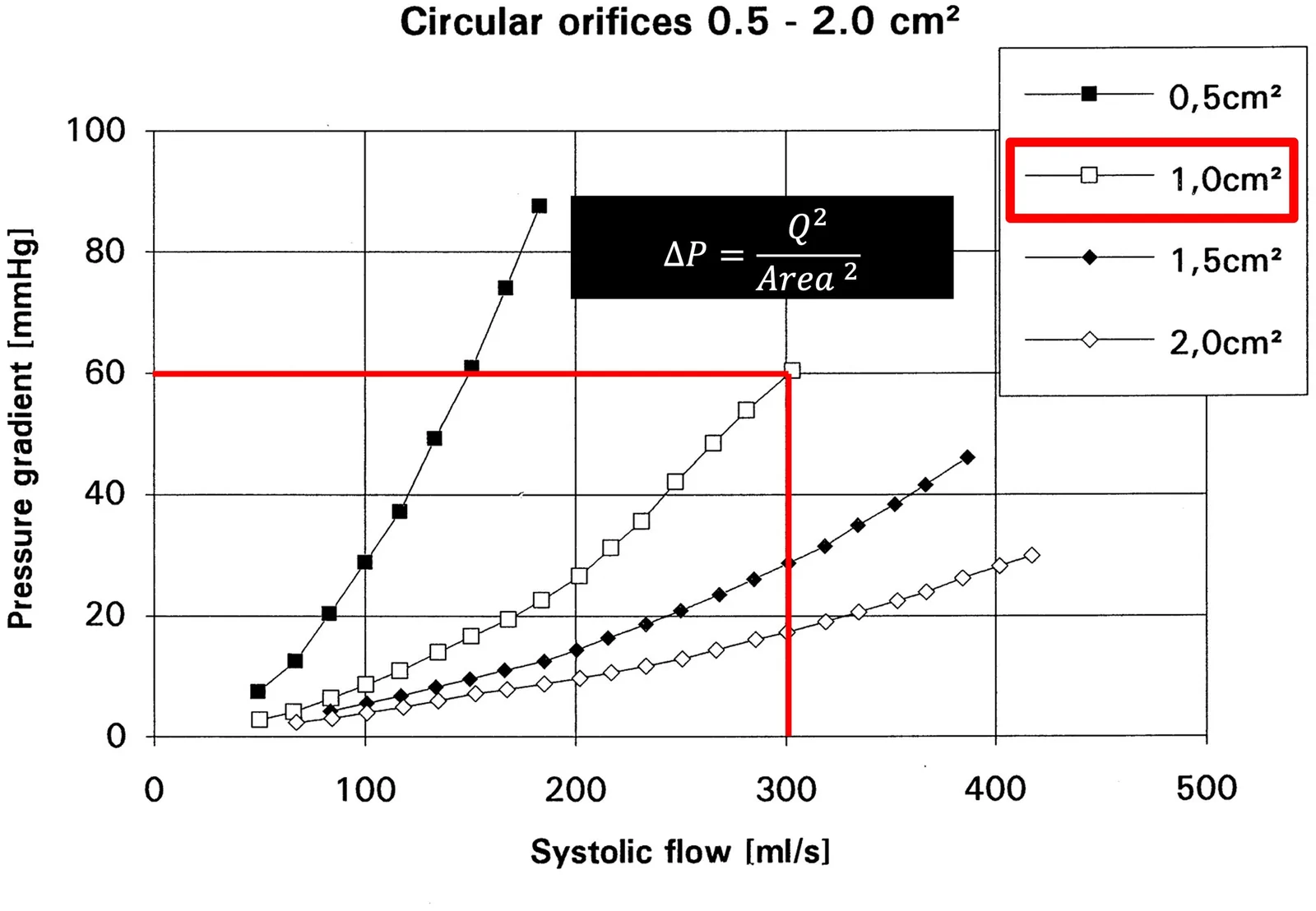
Curvilinear relation between flow rate and pressure gradients. The (extent of the) curvilinear relationship between flow rate (mL/sec) and pressure gradient is illustrated for different, fixed stenotic areas *in vitro* (adapted figure from reference,^26^ with permission). Q denotes flow (rate). Combining the Gorlin equation with Ohm’s law yields the equation $\Delta P=\frac{\;flow^{2}}{area^{2}}$. The red lines indicate the pressure gradient at a flow rate of 300 mL/sec when the fixed orifice is 1 cm^2^.

Pressure gradients are inherently dependent on valve area, flow (rate), and resistance, as expressed by Ohm’s law. When merging Ohm’s equation (flow x resistance = pressure gradient) with the Gorlin formula (for Gorlin equation, see *Figure 6*), the resulting equation demonstrates that Pmean is proportional to the square of flow divided by the square of a fixed valve area, illustrating a non-linear but variable relationship between flow and pressure gradients in AS with fixed AVA, as illustrated by an *in vitro* study in *Figure 7*.^27^ In fact, this is also reflected in the SB equation, where pressure gradient is proportional to the square of velocity (*Figure 1*, bottom right). However, because the AVA is often flow-dependent *in vivo*, the pressure–flow relation frequently adopts non-quadratic shapes in clinical practice, including linear relations.^14,28^ Albeit, a peak jet velocity of > 4 m/se at rest is specific for severe AS but lacks sensitivity, as velocities < 4 m/sec are frequently observed in patients with low-flow severe AS. Conversely, velocities > 4 m/sec may be occasionally encountered in moderate AS during high-flow conditions, such as tachycardia, anaemia, or fever.^3^ Therefore, pressure gradients should not be used in isolation to grade AS severity; rather, less flow-dependent measures, such as AVA measured with the continuity equation, should be integrated into the overall assessment. The continuity equation, although fundamental to the assessment of functional AVA in AS, bears theoretical and practical pearls and pitfalls (*Figure 6* and *Table 2*). Notably, when assessing AVA via the continuity equation, LVOT-based stroke volumes correlate best with cardiac magnetic resonance-based stroke volumes when cross-sectional areas are obtained within 2 mm of the annulus compared to more distant cross-sectional areas.^29^

**Table 2:** Continuity equation (‘what goes in must come out’): pearls and pitfalls

Pearls
*Validated in clinical studies and experimental studies
*Continuity equation-based valve area calculation is valuable for the prediction of clinical outcomes and decision-making
*Measures the functional area (EOA), which is most physiological
*Feasible in majority of patients
Pitfalls
*Assumes incompressible fluid
*Cannot be applied in case of ‘bypass’ circuits (example in *Figure 16*)
*Requires measuring three components of equation, each prone to error
*Assumes circular shape of estimated area using equation: area = π×r^2^
*Requires correct positioning of PWD sample to obtain VTI to calculate volume
*Affected by factors that distort the laminar flow profile or result in tandem stenoses
*Proper estimation of (stroke) volume is challenging with highly dynamic orifices, as occurs in LVOTO
*Continuity equation measures functional area (EOA), causing potential discrepancy with anatomical area
*Valve area depends on flow rate (under and overestimation)

From a haemodynamic perspective rooted in Ohm’s law, the assessment of AS raises the question of the minimum transvalvular flow rate required to reliably confirm true severe disease. In fact, flow rate can be considered as a surrogate for the kinetic energy ($E_{k}=\frac{1}{2\;}\;mv^{2}$) required to open the aortic valve, where *m* reflects the blood mass or volume with *v* being its velocity, the latter having major impact on the kinetic energy due to the squared relation. Flow rate can be calculated as LVOT stroke volume divided by left ventricular ejection time (ET). In AS, a flow rate of ≥210 mL/s has been proposed as a threshold above which valve area measurements are more reliable at rest,^30^ as this flow rate ‘threshold’ is sufficient to approximate full valve opening and reflect true stenosis severity. In such cases, additional testing with dobutamine stress echocardiography may not be necessary.^31^ The concept of projected AVA in discordant AS defined by a Pmean <40 mmHg and AVA <1.0 cm^2^ further refines this approach by normalizing AVA to a standardized reference flow rate of 250 mL/s during dobutamine infusion.^32^ Projected AVA has consequently been recommended as the preferred reference parameter over peak AVA.^33^ However, calculation of projected AVA may not be feasible if flow rates cannot be augmented by > 15%,^34^ and flow rate to AVA curves should be obtained through multipoint analysis to ascertain linear relationships in individual patients to allow reliable AVA projections. Finally, the use of dobutamine in classical low-flow, low-gradient AS can be complemented with the use of computed tomography to assess aortic valve calcification. While both modalities provide diagnostic information, each has distinct strengths and limitations that must be considered in clinical decision-making.^35–37^

In complex haemodynamic scenarios, such as mixed valve disease^38^ with moderate AS and at least moderate AR, the MB equation should theoretically be applied when LVOT velocities exceed 1.5 m/sec.^1,39^ Even without this correction, peak transaortic velocity remains prognostically informative as it reflects the overall haemodynamic load imposed on the LV.^40^ Similarly, in patients with discordant high-gradient AS, also referred to as pseudo-moderate AS (Pmean > 40 mmHg and AVA > 1.0 cm^2^), and unexplained high stroke volume, SB-assessed peak gradients retain prognostic significance.^41^

A challenging haemodynamic situation occurs in patients with combined AS and dynamic LVOT obstruction (LVOTO), i.e. serial obstruction with high pre-stenotic velocities. Here, high pulse repetition frequency (HPRF) Doppler time-aligned with peak AS velocity can be used to estimate maximal AS gradients employing the MB equation if v_1_ > 1.5 m/sec, with careful positioning of the sample volume <1 cm from the aortic valve.^42^ In contrast to conventional PWD, in HPRF Doppler, the system sends multiple pulses before all echoes from previous pulses return, which increases the PRF, allowing measurement of higher blood velocities with less aliasing, but at the expense of range specificity (depth accuracy).^42^ Because echocardiographic estimation of LVOT-based stroke volume is flawed by significant dynamic changes in outflow tract geometry and potential aliasing when using PWD, volumetric stroke volume measurements, derived from 2D or 3D imaging in the absence of more than mild MR and divided by the VTI across the aortic valve, may provide a more reliable approach in tandem dynamic and fixed obstruction.^42,43^ However, for complex cases as is the case for tandem stenosis, a multiparametric and multimodality approach is recommended.^42,44^ This includes valve area planimetry using cardiac magnetic resonance imaging, transoesophageal echocardiography, or computed tomography, with the latter incorporating aortic valve calcium scoring.^42,44^

#### Shape of the aortic stenosis continuous-wave Doppler signal

**Figure 8:**
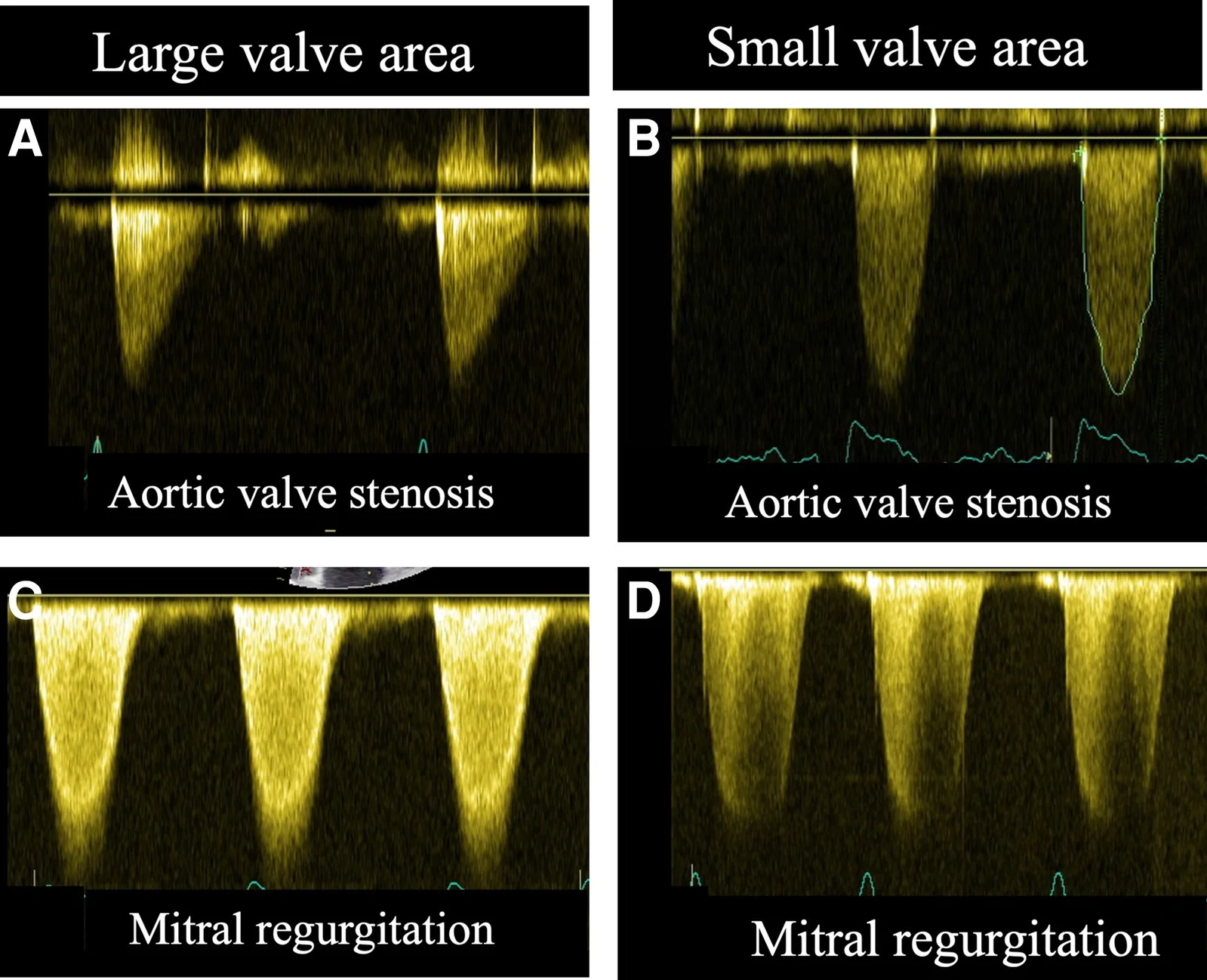
Impact of valve area on the continuous-wave Doppler shape. Impact of the stenotic or regurgitant orifice area on the CWD signal shape in AS (*A* and *B*) and MR (*C* and *D*). In general, a larger area generates an early peaking and triangular signal (*A* and *C*), whereas a relative smaller area generates a more parabolic, midsystolic peaking Doppler signal (*B* and *D*).

The relationship between Vmax and Vmean of the AS CWD signal is determined by the CWD shape, and the respective velocity ratios have been used to address haemodynamic AS severity (similar to TR as described below), but this ratio did not outperform Pmean with respect to clinical outcomes.^45^ In moderate AS, the CWD waveform often appears triangular and mostly exhibits early systolic peaking (*Figure 8A*). By contrast, in many patients with severe AS, the Doppler envelope assumes a more parabolic shape, characterized by delayed midsystolic peak velocities (*Figure 8B*), reflecting the delayed carotid pulse in severe AS (‘pulsus tardus’). The stronger and prolonged force development in severe AS is governed by the Anrep effect and concentric left ventricular hypertrophy, resulting in a net increase of actin–myosin cross bridges formation in order to overcome the increased afterload.^23^ The delayed midsystolic velocity peak reflects the underlying ejection haemodynamics and can be quantitatively assessed using the acceleration time (AT).^46^ In native AS, AT > 94 ms has been associated with severe disease, although with only moderate sensitivity (71%) and specificity (81%).^38^ In prosthetic valves, a higher threshold is used, with AT >100 ms suggesting severe obstruction.^47–49^ A similar AT cut-off (>100 ms) has also been proposed in classical low-flow, low-gradient AS to help differentiate true from pseudo-severe stenosis, with a sensitivity of approximately 77%.^50^ Because AT is influenced by factors such as heart rate and loading conditions, normalization to ET using AT to ET ratio has been proposed. In severe AS, both AT and ET tend to increase due to afterload-induced changes in left ventricular performance,^23^ but the net effect is an increased ratio in severe AS. Acceleration time–ejection time thresholds of greater than 0.32–0.35 have been shown to provide diagnostic and prognostic value, specifically in severe asymptomatic AS^51^ and in paradoxical low-flow, low-gradient states.^47–49^ Importantly, this ratio reflects not only valve severity but also left ventricular function and loading conditions. For example, high-flow states, such as at least moderate aortic regurgitation, may shorten AT despite severe stenosis, while systemic hypertension may prolong ET and shorten AT, leading to pseudo-normalization of the ratio.^49,52^ Atrial fibrillation presents particular challenges in assessing the AT/ET ratio due to beat-to-beat variability in flow and pressure gradients. In some studies, averaging measurements over five consecutive beats has been proposed, but this approach is valid only when the results are reproducible and consistent across cycles.^49^ Accordingly, the ratio should be interpreted as an integrative haemodynamic index rather than a standalone measure of valve severity, with careful consideration of potential confounders.

**Figure 9:**
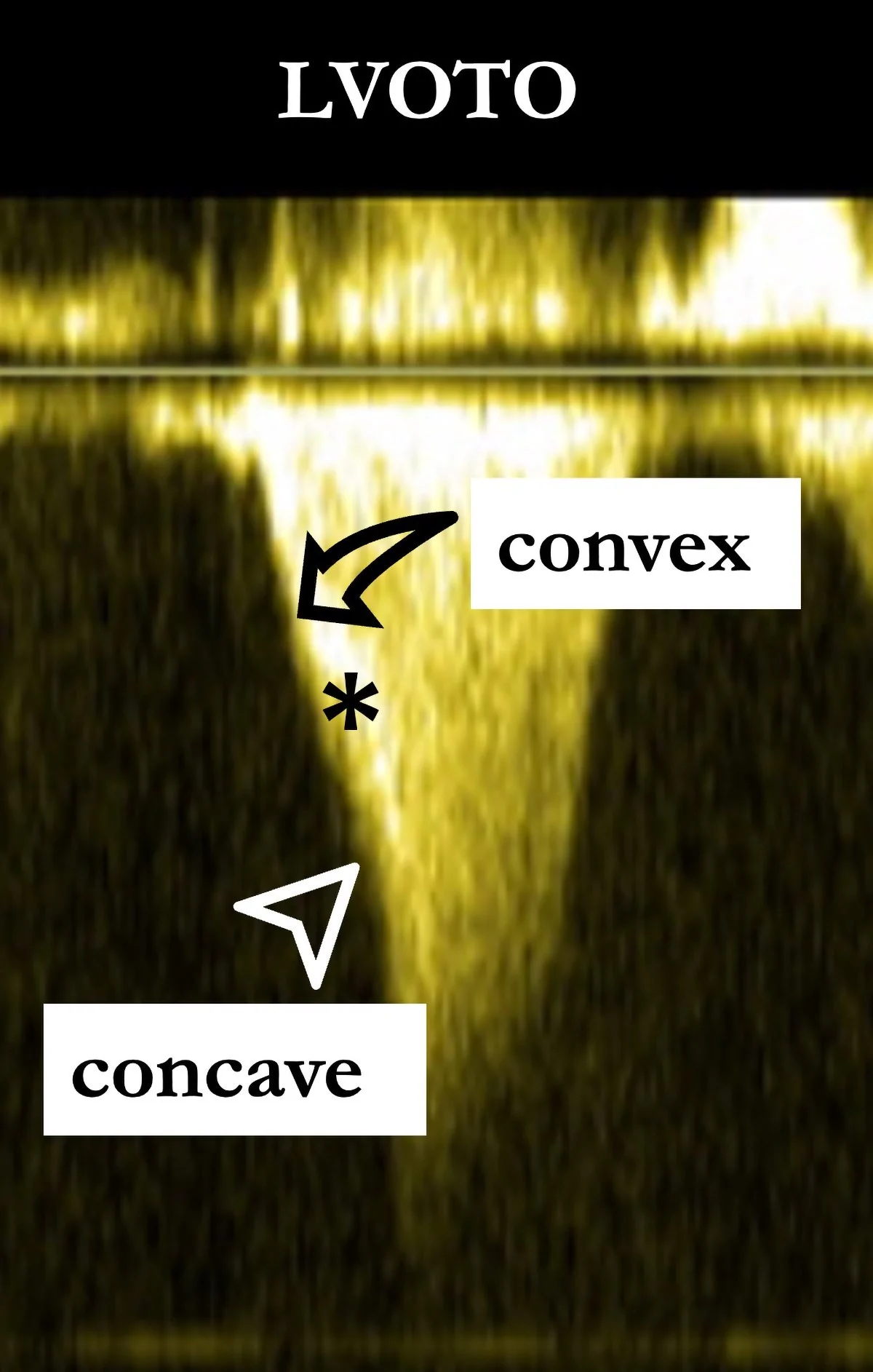
Dagger-shaped signal in left ventricular outflow tract obstruction. In patients with SAM-LVOTO, an initial ‘convex’ signal with low flow (black arrow) is followed by a more concave velocity spectrum (white arrowhead) starting at the inflection point (indicated by the asterisk), resulting in late systolic peak velocity.

Examination of the spectral Doppler envelope across the LVOT allows differentiation between fixed and dynamic obstruction and provides critical haemodynamic insights.^53–55^ Continuous-wave Doppler interrogation in fixed stenosis typically produces a rounded, parabolic envelope. However, a preserved aortic valve opening with a similarly rounded envelope may indicate subaortic membrane or supravalvular aortic narrowing.^54^ In patients with dynamic LVOTO due to systolic anterior movement (SAM) of the mitral valve, the CWD initially exhibits a convex contour reflecting early systolic flow in the LVOT, prior to the dynamic obstruction event (*Figure 9*).^53,56,57^ In fact, SAM occurs before onset of flow in the LVOT due to drag of the mitral valve leaflets towards the LVOT by the abnormal subvalvular apparatus.^56^ As systole progresses and SAM increases, the CWD envelope contour transitions to a concave contour at the inflection point, representing the onset of dynamic obstruction and resulting in a typical dagger-shaped Doppler signal (*Figure 9*).^53^ Patients with hypertrophic cardiomyopathy and mid-ventricular or subapical obstruction associated with apical aneurysm formation may typically demonstrate a midsystolic signal void followed by paradoxical diastolic flow on CWD interrogation.^58^

#### Invasive vs. Doppler assessment of aortic stenosis

**Figure 10:**
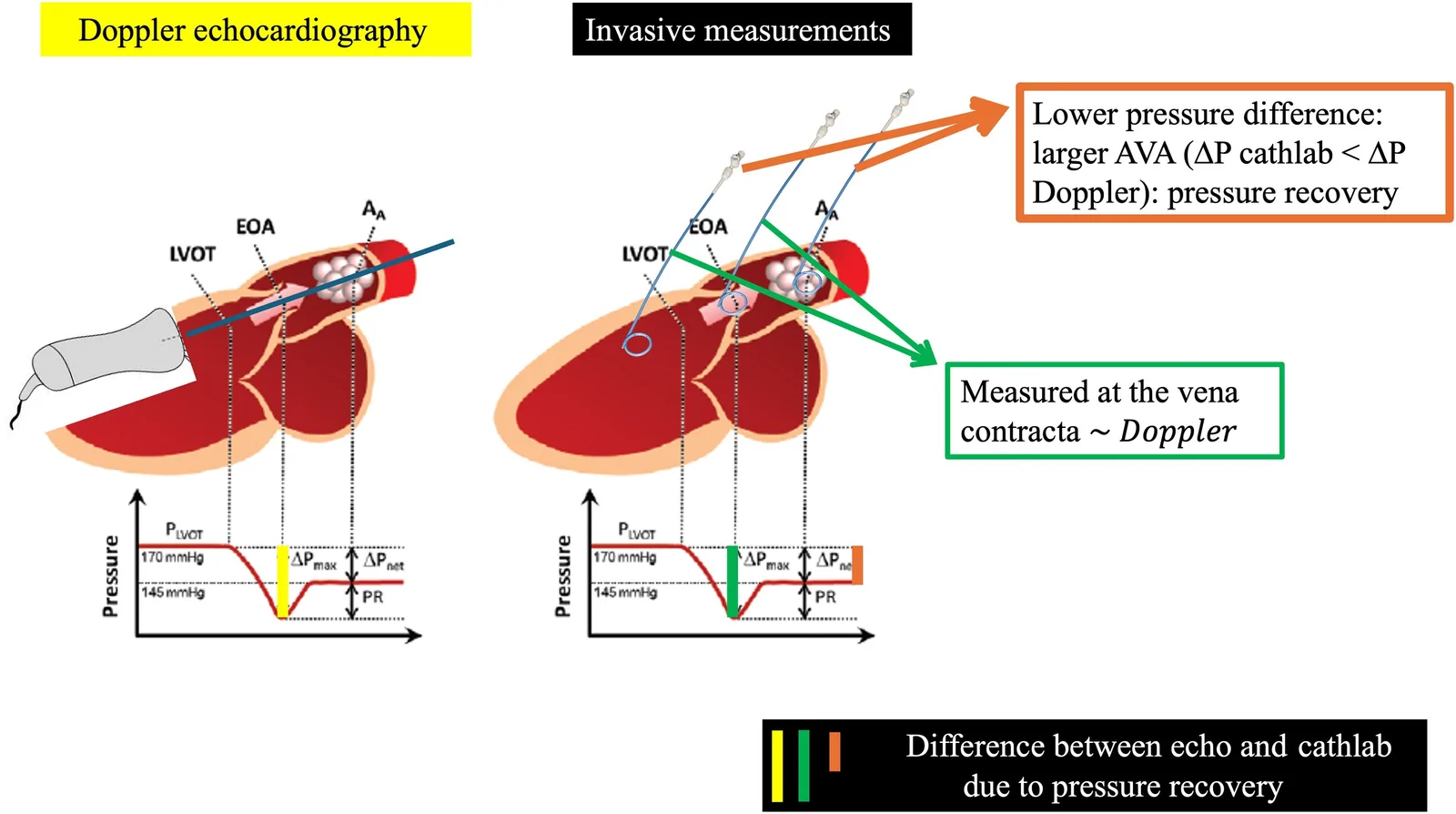
Illustration of pressure recovery in aortic valve stenosis. Doppler echocardiography in AS reveals the maximal, instantaneous pressure gradient at the vena contracta (yellow bar), which corresponds to the invasive pressure gradient when the pigtail catheter would be positioned at the vena contracta (green arrows and green bar). When the pigtail catheter is pulled back into the ascending aorta, the pressure difference (brown bar) between the intracardiac and aortic pigtail catheter (brown arrow) is much less pronounced in this example, due to pressure recovery in the ascending aorta. Figure modified from reference,^60^ with permission. A_A_, aortic area at the sino-tubular junction; AVA, aortic valve area.

Despite overall concordance between invasive and Doppler-derived gradients, discrepancies are well documented in AS, as summarized in *Table 3*.^59^ Invasive measurement of AS severity traditionally compares peak left ventricular pressure to peak aortic pressure. However, this ‘peak-to-peak’ approach is not physiological because peak LV and aortic pressures occur at different times, particularly in severe AS characterized by delayed aortic upstroke. The error may be further amplified during pull-back pressure recordings due to beat-to-beat variability, but this can be circumvented by dual-lumen catheters that allow simultaneous pressure recordings in the LV and aorta. Since Doppler-derived gradients reflect the instantaneous peak velocity between the left ventricle (LV) and aorta, they are inherently greater than peak-to-peak invasive gradients.^59^ Finally, another important source of discrepancy between echo Doppler and invasive AS assessment in clinical practice is the phenomenon of pressure recovery, the partial reconversion of kinetic energy to potential energy downstream of a stenotic lesion (*Figure 10*).^24,61^ Indeed, CWD cannot account for pressure recovery and only captures the highest pressure drop at the vena contracta, explaining why CWD yields higher gradients compared to invasive measurements in the context of significant pressure recovery. As a consequence of pressure recovery, according to the Gorlin-based AVA equation in *Figure 6*, a lower invasive MPG will yield a larger AVA compared to echo Doppler. In a theoretical, idealized system without energy losses, complete pressure recovery would occur at identical tube diameters before and downstream of the stenosis. In vivo, however, pressure recovery is always partial and is influenced by factors such as stenosis severity, flow turbulence, and aortic diameter.^62^ Pressure recovery is most pronounced in less severe AS and in patients with a small ascending aorta (<30 mm). It has been suggested that in such cases, invasively measured gradients may better reflect true left ventricular afterload.^61^ Therefore, some investigators have proposed to ‘adjust’ the AVA for pressure recovery, using the so-called energy loss index (*Figure 6*). Although this correction may reclassify AS severity in selected cases, it has not been widely adopted in routine clinical practice, as it adds computational complexity and its incremental value over established methods remains to be clearly demonstrated.^63,64^ Conversely, patients with bicuspid aortic valves and dilated ascending aortas often exhibit disturbed flow with increased viscous and turbulent energy loss, which may impose an additional haemodynamic burden on the LV not captured by Doppler-based gradients.^65^ Whether assessment of post-stenotic kinetic turbulence,^66^ incorporation of flow patterns, or considering pressure losses in significant aorta dilation in the haemodynamic assessment of AS severity may improve prognostic accuracy remains to be explored.

**Table 3:** Discordance between echocardiographic and invasively calculated aortic valve area

*Gorlin area matches the anatomical aortic valve area and incorrectly assumes negligible flow contraction
*Doppler inherently considers flow contraction and derives the functional area of the valve orifice
*Pressure gradient (beat-to-beat and peak-to-peak) between echo and catheterization uses different approaches
*Pressure recovery may be relevant with a small ascending aorta diameter and in valve prostheses
*Invasive cardiac output measurement by thermodilution has inherent challenges and limitations
*Pressure measurement errors can occur during invasive assessment
*Echo measurements have inherent challenges and limitations (LVOT, PWD, Doppler signal acquisition, etc.)

Pressure recovery also occurs in transcatheter and surgical prostheses, particularly bileaflet mechanical valves, where Doppler may overestimate transvalvular gradients.^13,67^ In these devices, manufacturer reference values already account for this effect.

Doppler-derived AVA, based on the continuity equation, is typically smaller than the invasively calculated Gorlin area, which itself an approximation of the anatomical AVA (*Figure 6*). The Gorlin area requires meticulous invasive measurements of all its components (*Figure 6*), and apart from several other limitations of the Gorlin equation,^68^ it neglects flow contraction effects which additionally explains higher invasive AVA compared to Doppler-derived functional AVAs.^25^ Similarly, planimetric measurements by echocardiography, computed tomography, or magnetic resonance imaging yield larger anatomic AVA compared to the Doppler-assessed functional AVA (*Figure 6*), and do not directly correspond to functional haemodynamic severity, since contraction of flow is ignored.^69,70^ Contraction of flow lines at the stenosis may be as high as 40% of the anatomical AVA, and flow contraction is affected by valve geometry (e.g. funnel-shaped promotes less flow contraction than a flat AVA geometry^71^), blood viscosity, eccentricity of jet stream, and flow rate.^25,72,73^ Finally, Doppler-based areas are time-integrative, whereas in planimetry, the largest orifice is considered. The optimal anatomic cut-off to define severe AS remains debated,^74^ although some authors suggest a threshold of 1.2 cm^2^.^70^ In a recent computed tomography study, an anatomical AVA cut-off ≤ 0.95 cm^2^ optimally defined severe AS in tricuspid aortic valves and ≤ 1.08 cm^2^ for bicuspid valves, although the latter population was limited in that study.^75^

Although Bernoulli’s principle assumes a short, laminar flow convergence and steady velocity profile, these assumptions are frequently violated in contemporary percutaneous aortic prostheses, which vary in design and induce complex post-valvular flow.^13^ Consequently, significant discordance between Doppler- and catheter-derived mean gradients is frequently observed following transcatheter valve implantation.^13^ In fact, Doppler-derived mean gradients do not reliably predict outcomes after transcatheter aortic valve replacement, whereas time-based indices such as AT and especially AT/ET appear more robust.^76^

#### Dimensionless index in aortic stenosis

**Figure 11:**
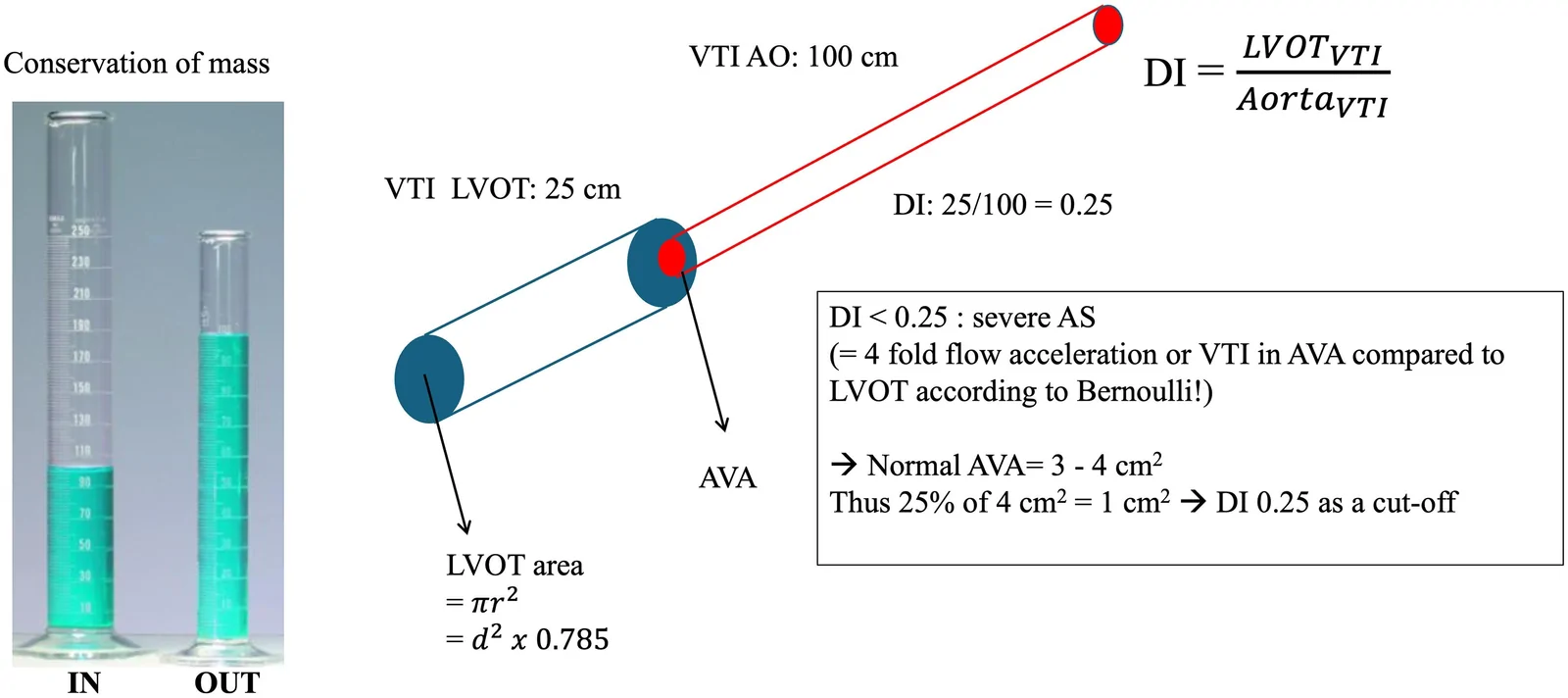
Illustration of the dimensionless velocity–time integral index. The continuity equation, based on the conservation of mass, inherently assumes a constant relation between the VTI (or peak velocity) of LVOT and the VTI of a fixed AS area (AVA). DI denotes dimensionless index.

The DI is a ratio routed in the conservation of mass principle, reflects the relative velocity acceleration across a stenotic orifice and provides a simplified measure of AS severity (*Figure 11*).^77^ It is calculated from velocities measured in the LVOT using PWD and across the aortic valve using CWD, without requiring measurement of the outflow tract diameter.^78,79^ Both peak velocity and VTI measurements can be used to calculate the DI, although one study suggested that peak velocity measurements demonstrate greater reproducibility.^80^ A VTI-based DI cut-off of 0.25 for severe AS is commonly used,^81^ which corresponds to a quarter of the normal AVA, which is usually between 3 and 4 cm^2^,^79^ thus yielding an AVA ranging between 0.75 and 1.0 cm^2^ as a reference. Thus, while the DI is independent of absolute LVOT size, it may still vary when AS severity is defined by AVA. For example, one study demonstrated that an AVA of 1 cm^2^ corresponded to DI values of 0.22, 0.29, and 0.36 in patients with large, average, and small LVOT diameters, respectively, with the smallest diameters typically observed in elderly female patients.^82^ In other words, the DI may still be dependent on the LVOT dimension. The prognostic utility of the DI has been well established across a broad spectrum of AS populations. In patients with low-flow, low-gradient AS, a DI of <0.25 identifies patients at a higher risk of death.^83,84^ Similarly, in patients with surgical aortic valve prostheses, values of 0.35 or less are associated with adverse prognosis.^85^ In the context of self-expanding valves for transcatheter aortic valve replacement, a DI of <0.50 has been shown to be prognostically relevant, while similar association has not been demonstrated in balloon expandable valves.^86^ Finally, the DI is an additional tool to discriminate pseudo-AS from true AS during dobutamine stress testing.^32,87^

### Aortic regurgitation

The CWD signal in AR is used to estimate AR severity using the PHT method and via the proximal isovelocity surface area (PISA)-based effective regurgitant orifice area (EROA) calculation by using the peak velocity of the Doppler spectrum, but several limitations regarding the PISA method should be considered.^88–90^ In addition, accurate acquisition of the AR CWD signal can be challenging, particularly in the presence of eccentric jets, leading to unreliable PISA-derived EROAs and PHT measurements due to Doppler misalignment. Even when the AR CWD signal is optimal, PHT has the lowest sensitivity among commonly used methods for identifying significant chronic severe AR, whereas its specificity to detect moderate–severe or severe AR was 85% in one study.^91^ One of the limitations in using the PHT arises from the incorrect assumption that pressure decay is determined solely by the (fixed) EROA. In reality, the rate of deceleration of AR velocities reflects the equilibration of diastolic pressure gradients between the aorta and LV. Therefore, reduced LV diastolic compliance may significantly reduce PHT, hence overestimating AR severity.^89^ Even in non-severe AR, a noncompliant LV may significantly shorten PHT, especially in the acute or subacute settings. Yet, in clinical practice, a short PHT < 200 msec mostly hints towards severe AR until proven otherwise and especially in acute severe AR cases where the LV has no time to adapt.^78^ Conversely, a dilated, compliant LV may exhibit prolonged PHT values despite severe AR. In addition to LV myocardial properties, PHT may also be affected by afterload, i.e. systemic vascular resistance and blood pressure. Thus, in many patients, PHT is merely a marker of the haemodynamic consequences of AR rather than the regurgitant volume (RegVol) itself. Finally, heart rate can influence PHT in AR, as higher heart rates are associated with a steeper regurgitant slope.

## Mitral valve disease

### Mitral regurgitation

Peak velocities in MR are used to calculate EROA using the PISA method^92^ (PISA EROA = flow rate/Vmax of the MR CWD signal), but in patients with mitral valve prolapse and SAM-mediated MR, peak velocities may be difficult to assess due to jet eccentricity and therefore potentially confound reliable PISA-based EROA measures. Peak velocity values are in general lower in functional MR (FMR) compared to primary MR (PMR). In fact, peak velocities in PMR appear not to change significantly with increasing degrees of MR severity, whereas in FMR, this variability is more evident, which is due to LV systolic dysfunction and increased LA pressures.^5,93^ It is important to realize that for the same regurgitant volume, increasing jet velocity leads to a larger colour Doppler jet area due to higher jet momentum and where the momentum transfer from the high-velocity jet to surrounding RBCs in the left atrium (LA) increases the colour Doppler jet area.^94,95^

For atrioventricular valvular regurgitation, the hydraulic (orifice) equation is conventionally written as RegVol = EROA × VTI. Because VTI is determined by duration of the Doppler signal (t, time) multiplied by the square root of the mean pressure gradient (MPG), the hydraulic equation can be rewritten as RegVol = EROA × t × √MPG.^96^ The MPG is the mean LV–LA pressure gradient over the atrioventricular valve. Thus, although increasing LV–LA pressure gradients do increase the RegVol for a given EROA, the square root relation with the degree of RegVol suggests that RegVol becomes most apparent when large changes of MPG (and thus blood pressures) occur.^97^

#### Shape of the mitral regurgitation continuous-wave Doppler signal

**Figure 12:**
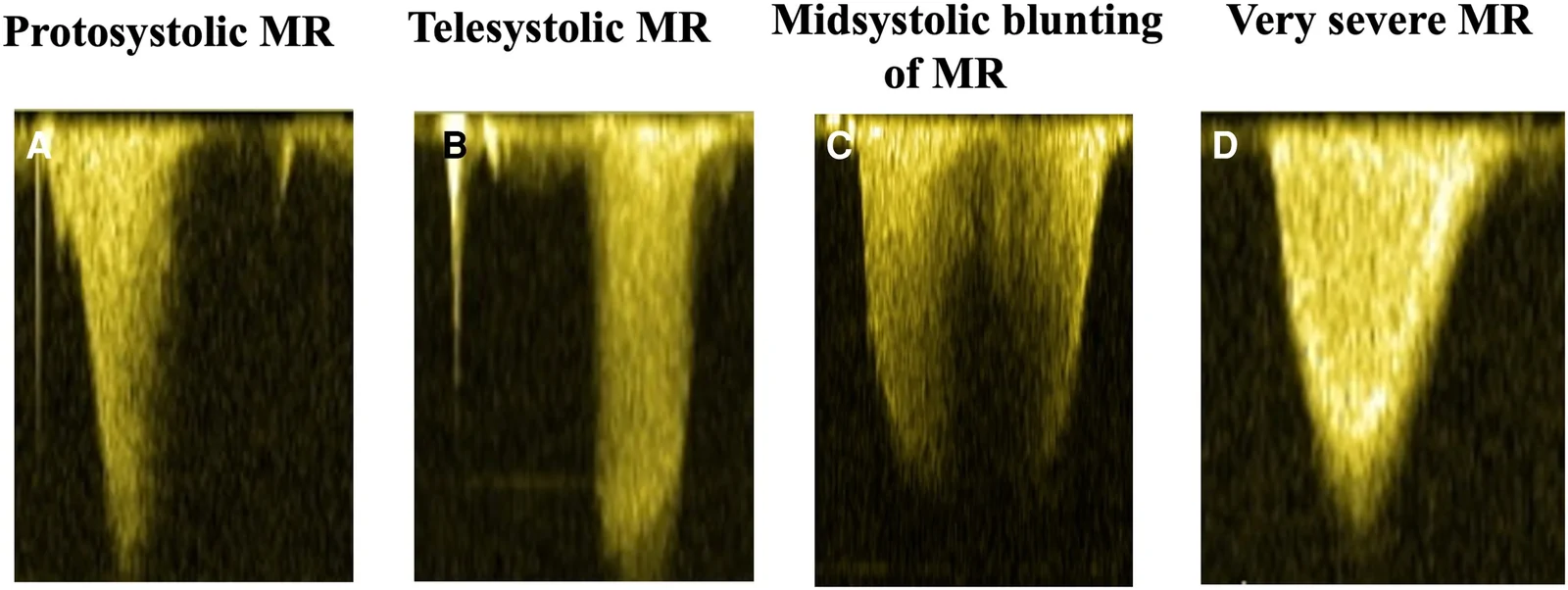
Timing and regurgitant duration in mitral regurgitation. (*A*) shows a protosystolic MR flow in a patient with atrial fibrillation and (*B*) a telesystolic signal in mild mitral valve prolapse with a centrally oriented jet. (*C*) In mild FMR, there is significant blunting of midsystolic regurgitation due to mitral valve closure. In this situation, tracing the entire envelope will yield a too large VTI and overestimation of RegVol when using the PISA method. In (*D*), a patient with very severe FMR is shown with a triangular and very intense Doppler signal with small VTI.

The shape of the CWD signal is mostly parabolic in MR (*Figure 8D*), except in cases of non-holosystolic MR, such as occurs in protosystolic MR or in telesystolic MR in patients with mitral valve prolapse (*Figure 12*). Although most non-holosystolic MR jets are non-severe,^98^ the cumulative RegVol of multi-scallop non-holosystolic MR jets may eventually become severe with RegVol > 60 mL, and thus these patients may require a volumetric 2D or 3D RegVol assessment and/or cardiac magnetic resonance^5,99^ More pronounced triangular CWD shapes are unfrequent but may occur in MR, especially in acute severe MR, and in patients with very severe FMR. In the latter patients, this CWD waveform is due to large EROA, poor LV function, and elevated left atrial pressures (*Figure 8C*).^93^ Along with the more triangular shape, these Doppler signals exhibit lower peak velocities and lower VTI values.^93^

**Figure 13:**
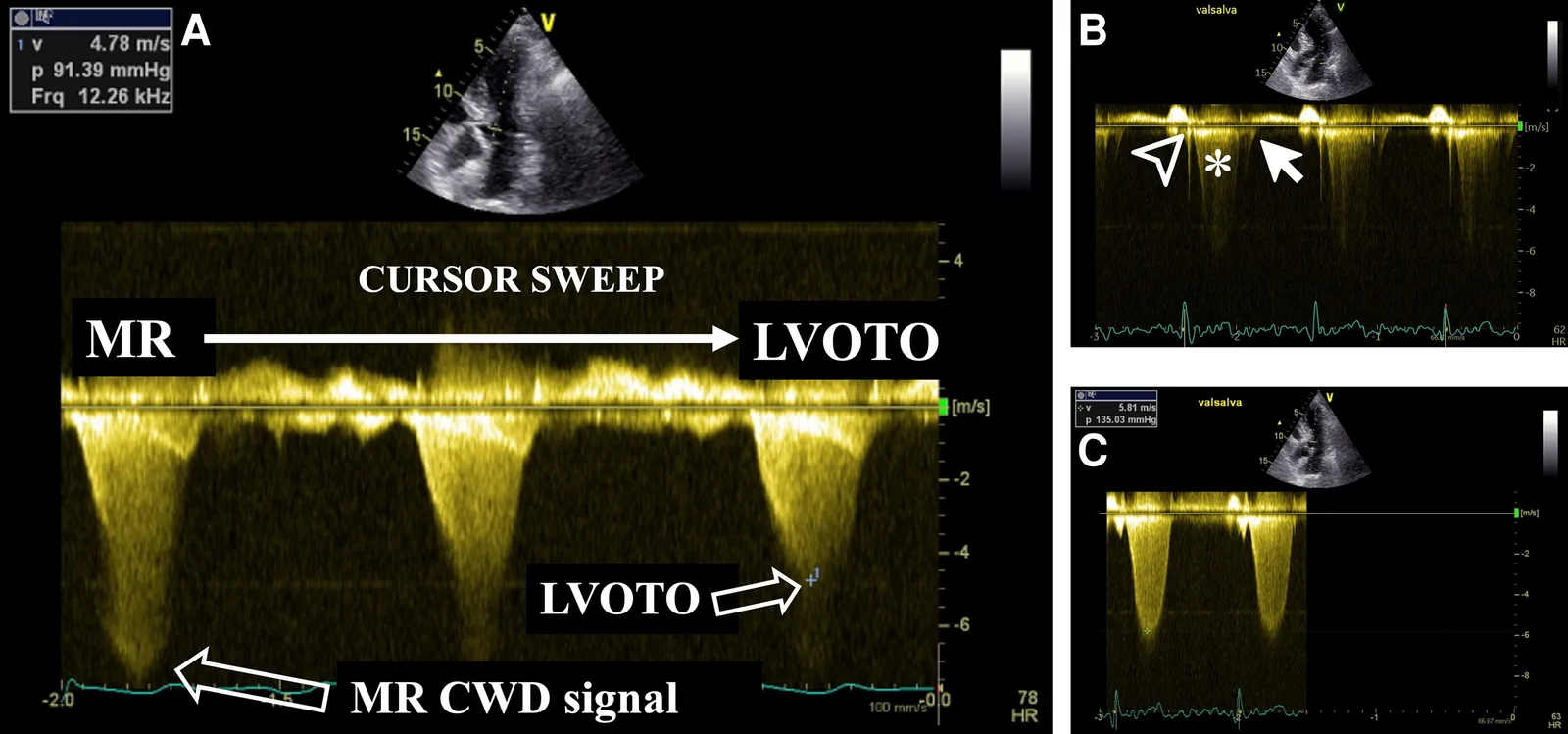
Mitral regurgitation and left ventricular outflow tract Doppler signal. The MR signal has an initial slower acceleration followed by a typical steep–straight acceleration phase (Panel *A*, left signal). The MR signal can be excluded by sweeping the cursor towards the LVOT, resulting in the more late-peaking LVOTO dagger-shaped signal (right signal). In B, the arrowhead and arrow show the start and end of the MR signal, respectively, with part of the LVOTO signal indicated by an asterisk. In C, the MR signal is excluded from the LVOTO signal when sweeping more towards the LVOT.

A challenging clinical scenario may often occur in patients with both LVOTO and MR, in particular when trying to discriminate the MR CWD signal from the dynamic LVOTO CWD signal. It may be helpful to search for the MR signal first by sweeping the cursor towards the mitral valve and then sweeping back to the LVOT to capture the typical mid-to-late systolic peaking, dagger-shaped LVOTO signal (*Figure 13A*). The MR CWD in patient with LVOTO commonly has Vmax values > 5 m/sec and has higher velocities than the LVOTO signal, and it typically starts earlier and terminates later compared to the LVOTO signal (*Figure 13B*). Also, the MR CWD signal, after an initial curved acceleration phase, straightens out at midsystole compared to LVOTO which continues to show rightward curvature in midsystole (*Figure 13 A*, first Doppler signal). Thus, following the chase for MR & LVOTO signal, a careful look at timings and shapes may help in discriminating these signals. Finally, subtracting the systolic blood pressure from the estimated peak LV pressure as a raw estimate of the LVOTO peak gradient can be considered, i.e. (CWD peak gradient + peak LA pressure)—systolic blood pressure = peak LVOTO gradient. However, this ‘plausibility check’ may be flawed in case of suboptimal MR signal acquisition, when time to peak of the LVOTO and MR signal does not align, and confounded by errors related to assumptions of peak LA pressure are inherent.

#### The velocity–time integral of mitral regurgitation

**Figure 14:**
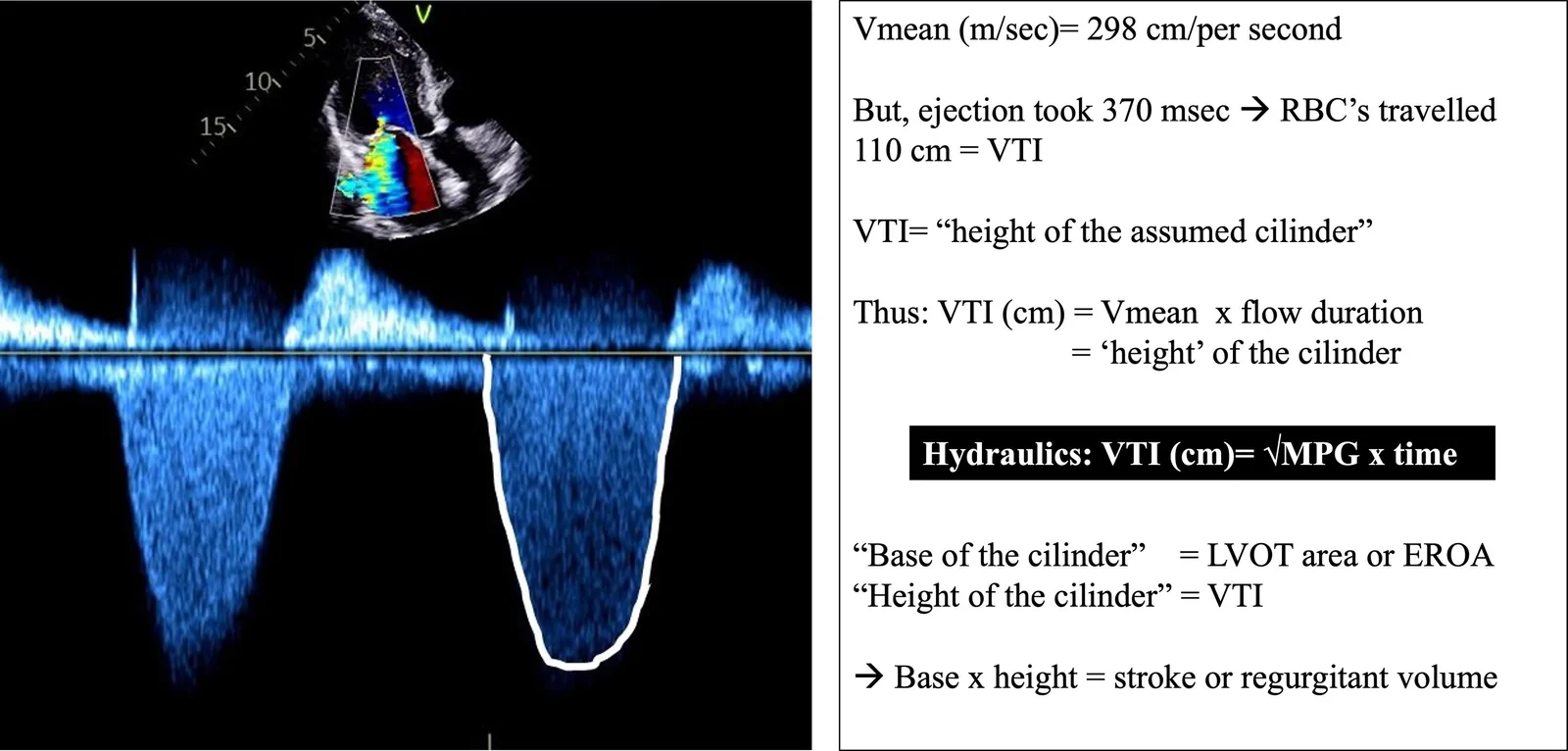
The velocity–time integral explained. The VTI can be calculated by correcting the Vmean of the CWD signal for the duration of the flow. It is expressed as cm and represents the average distance that the interrogated RBCs have travelled during the flow event. Mathematically, it represents the area occupied by the pixels in the CWD signal.

The VTI represents the sum of velocities at each time point during a single ejection or regurgitation period, corresponding to the area under the velocity curve^2^ (*Figure 14*). Assessing VTI values is less reproducible compared to peak velocities.^100,101^ Similar to all Doppler-based derivatives, the VTI itself is influenced by orifice area, the pressure gradient which itself depends on orifice area and ventricular contractility, and flow (rate).^93^ Practically spoken, the VTI represents the average distance travelled by RBCs during the observed event, expressed in cm for convenience. When multiplied by the corresponding ‘fixed’ cross-sectional area in cm^2^, it yields stroke volume or RegVol (in ml or cm^3^). In a closed, unbypassed circuit without shunts or regurgitation, stroke volume remains constant, reflecting the principle of conservation of mass. This principle forms the basis of the continuity equation, which is widely applied in haemodynamic assessment, including the calculation of AVA in AS (*Figure 6*) and EROA using PISA approach for MR, TR, or AR.^2,102^

In MR, VTI is used for the PISA method to calculate RegVol (PISA-RegVol = PISA-EROA × VTI). Conversely, in 2D/3D volumetric approaches, VTI of MR signal is used to calculate the EROA as EROA = RegVol/VTI. In cases of non-holosystolic regurgitation, it is essential to trace the most intense, clearly visible portion of the CWD signal to obtain the VTI (*Figure 12*).^5,78,103^ As a rule of thumb, RegVol are smaller in non-holosystolic MR jets, and single-frame EROA measurements such as those obtained with the PISA method may therefore overestimate the severity of regurgitation in such cases.^103–105^ In instances of marked midsystolic blunting of the regurgitant CWD signal, such as in mild or moderate FMR,^105–107^ accurate tracing of the VTI is challenging, and such signals generally indicate non-severe regurgitation (*Figure 12 C*).^105^ In these cases, as well as in patients with multiple regurgitant jets, a multiparametric echocardiographic approach using volumetric 2D or 3D methods is recommended; if uncertainty persists, multimodality imaging, such as cardiac magnetic resonance, may be considered.^99,105,108^

Because the VTI links EROA and RegVol, it can also be used as a consistency check for echocardiographic grading of MR or TR. For example, in FMR, patients with severely depressed LVEF and EROA in the severe range (≥ 0.4 cm^2^), a RegVol of 60 mL would require an average VTI of 150 cm (i.e. 60 cm^3^/0.4 cm^2^), which is unlikely to occur in this compromised haemodynamic situation,^109^ i.e. a lower VTI is expected here,^109^ indicating suboptimal assessment of EROA and RegVol.

In prosthetic mitral valves, a dimensionless ratio is calculated by dividing the transmitral VTI by the LVOT VTI. This index provides a measure of prosthetic valve function that is relatively independent of valve size.^67^ Evidence from clinical studies indicates that the dimensionless ratio is accurate in detecting both mechanical and bioprosthetic valve dysfunction. Importantly, this parameter is sensitive to both regurgitant and stenotic lesions: significant regurgitation increases forward flow through the mitral valve while reducing systemic forward flow, whereas significant stenosis increases the transmitral VTI due to the restricted orifice area. Consequently, the dimensionless ratio offers a useful, practical tool for assessment of prosthetic mitral valve haemodynamics.^67^

#### Continuous-wave Doppler signal intensity in mitral regurgitation

**Figure 15:**
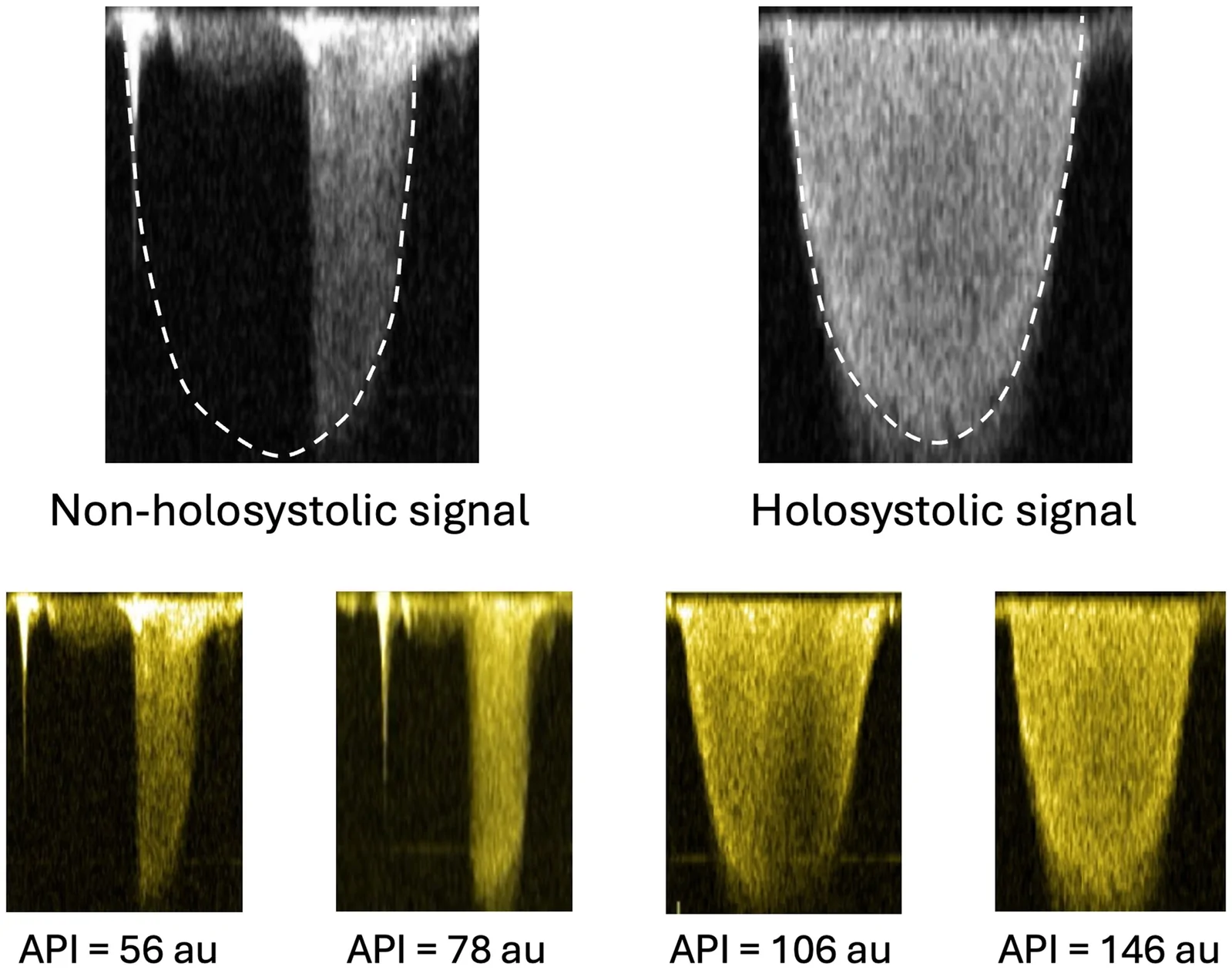
The average pixel intensity of the continuous-wave Doppler signal in mitral regurgitation. In the left upper panel, a parabolic tracing is traced, guided by the onset at the mitral closure click and extrapolated to the expected/estimated parabolic shape that includes the CWD signal to capture the entire systolic duration. Hence, the tracing includes the complete regurgitant flow variation in time; this tracing is more obvious for the holosystolic MR jet in the upper right panel. In the left lower panels, two telesystolic CWD signals typical for mitral valve prolapse are shown with relative low API values (56 and 78 au), whereas the lower right panels show two holosystolic MR jets with an API value of 106 au (moderate MR) and 146 au (severe MR), respectively (see also text for explanation).

The primary determinants of the CWD signal intensity are (i) the number of RBCs being interrogated, which reflects the regurgitant area and volume;^110–112^ (ii) the angle between the Doppler beam and the regurgitant jet; (iii) signal attenuation due to physical properties of the patients habitus; and (iv) machine settings and vendor-specific signal processing algorithms.^5^ The intensity of the CWD signal can be quantified using simple software by converting the red–blue–green format into a grayscale image. The CWD signal in holosystolic MR consists on average of 9000 pixels (for General Electric echo devices), and the intensity of each individual pixel ranges between 256 different shades of grey, from pure black [0 arbitrary units (au)] to pure white (256 au), which is a universal ranking convention in digital systems. By tracing the entire systolic cycle based on the MR CWD signal and/or mitral valve opening and closing clicks, the average pixel intensity (API) of the traced CWD envelope can be measured (*Figure 15*).^5,113^ Recent studies have demonstrated both diagnostic and prognostic value of the API method of the Doppler signal in MR.^5,113–117^ These translational data from *in vitro* studies towards clinical outcome studies have provided robust evidence that the API of the CWD signal in MR is mostly determined by the mitral regurgitant area and RegVol. By extension, similar to a cardiac magnetic resonance study,^104^ a more recent study demonstrated that the pixel variation score of the CWD signal also allows to capture and quantify flow variability during MR.^105^ A key advantage of the API and pixel variation score approach is that it integrates the entire systolic cycle, thereby avoiding the limitations inherent to single-frame grading methods, such as PISA-based EROA and 2D or 3D vena contracta width or area. Unlike these conventional methods, the pixel intensity approach does not rely on complex calculations, geometric assumptions, or correction factors.^5^ Despite its potential, the widespread adoption of this method is currently limited due to proprietary constraints, dependence on vendor-specific settings, sensitivity to physical properties of the imaging system, and the effects of beam angulation and attenuation. Yet, every single MR grading method has clear limitations,^78,88^ and specific pitfalls of the API approach can be addressed in future research,^115^ potentially allowing broader application. At present, international guidelines do not recommend the use of API method for routine grading of MR, but a recent machine learning AI algorithm to detect clinically significant MR included the intensity of the CWD MR signal.^117^

### Mitral stenosis

Mitral stenosis (MS) severity can be evaluated using direct anatomic 2D or 3D planimetry, particularly in rheumatic disease, or via indirect mitral valve area (MVA) methods, such as the continuity equation, PISA-derived MVA, or PHT-based MVA calculation, as well as by assessing the transvalvular MPG.^118–120^

**Figure 16:**
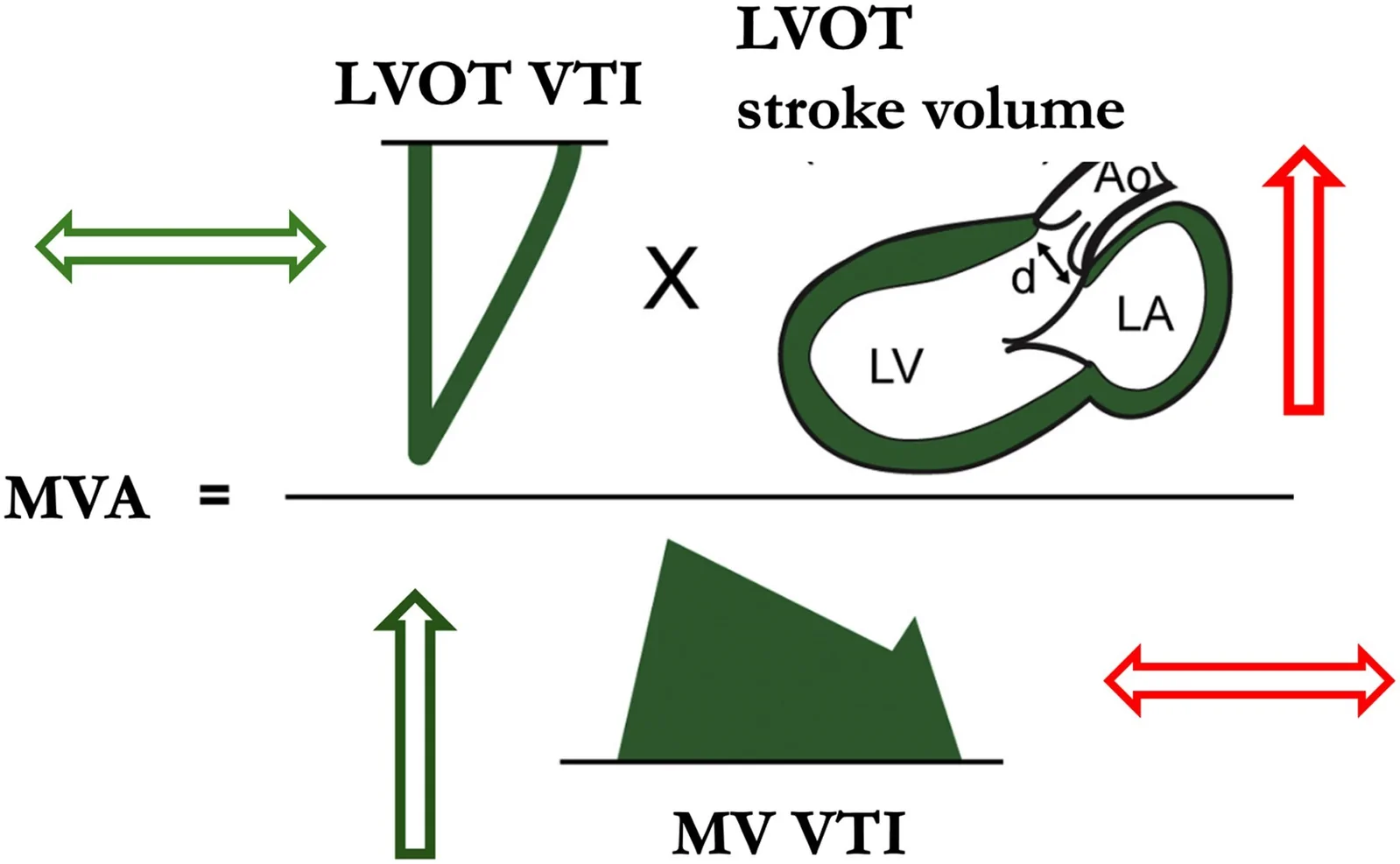
The continuity equation to assess mitral valve area in mitral stenosis. Underestimation of MVA may occur in concomitant ≥ moderate MR because it increases the denominator (MV TVI) (green arrow), whereas the numerator (i.e. the LVOT stroke volume) remains relatively constant (green bidirectional arrow). In concomitant ≥ moderate AR, MV TVI remains relatively constant (red bidirectional arrow), whereas LVOT–stroke volume increased (red arrow), eventually leading to overestimated MVA.

The VTI of mitral inflow is used in the continuity equation to calculate MVA in MS. However, several pitfalls can affect its accuracy. For example, concomitant MR can increase mitral inflow VTI,^121^ potentially leading to underestimation of MVA, whereas significant AR may result in overestimation of MVA by increasing forward aortic stroke volume (*Figure 16*).^39^ Continuous-wave Doppler-based PHT is routinely used to grade rheumatic MS severity. PHT-derived MVA is based in part on an empirical formula,^122^ but it generally performs well in clinical practice despite potential confounders such as atrial compliance and left atrial pressure.^123^ The feasibility and accuracy of the PHT method may be reduced in blunted or curved Doppler deceleration contours (‘ski slope’ deceleration pattern).^22^ In patients with combined MS and diastolic dysfunction with reduced ventricular compliance, or in those with combined MS and significant MR, PHT-based calculations may overestimate MVA.^121,124,125^ Because of these limitations, a multiparametric approach is recommended for grading MS severity. Three-dimensional valve area planimetry is preferred in rheumatic MS with commissural fusion, and it provides a flow-independent method.^120,126^ Assessment of stenosis severity in calcific MS is more complex. Doppler-based parameters, including MPG and continuity equation-derived MVA, may provide prognostic information in that case, especially in combined MR and MS.^127^ Anatomic evaluation using three-dimensional echocardiography or cardiac computed tomography also plays an important role, particularly when intervention is being considered.

Finally, because the MPG is strongly influenced by heart rate and stroke volume in MS, an equation has been proposed to estimate normalized gradients that account for these variables and improve the overall diagnostic accuracy.^128^

## Tricuspid valve disease

### Tricuspid regurgitation

#### Estimation of pulmonary pressures

Careful Doppler alignment and evaluation of the full TR velocity spectrum are essential for reliable assessment of TR velocities (*Table 4*). An important caveat in the assessment of TR peak pressure gradients using CWD is the concomitant presence of pulmonary stenosis (PS) or RVOT obstruction. In such cases, the obstruction-related gradient is superimposed on the TR-derived pressure gradient. Accordingly, the PS (or RVOT obstruction) gradient must be subtracted from the TR gradient to accurately estimate pulmonary artery pressure. Because trace TR often yields protosystolic signals or other CWD signal dropouts, reliable measurement of the TR jet velocity may be inaccurate or impossible in a substantial proportion of these subjects. In fact, in one study, no correlation was found between echo-based pulmonary pressure estimates and invasive pulmonary pressure measures in patients with trace TR.^129^ Ultrasound enhancing agents (UEAs) do not increase TR severity, but they simply increase signal-to-noise ratio, therefore improve delineation of the CWD envelope, and increase feasibility and accuracy of TR velocities in trace or mild TR. However, over-gain and excessive dose of UEAs can artificially widen the Doppler signal (spectral blooming), causing potential overestimation of CWD velocities.^9^

**Table 4:** Factors contributing to discrepancy between echocardiographic estimates and catheter-based measurements of pulmonary pressures

*Suboptimal Doppler signal quality (> 50% in advanced lung disease)
*Nonsimultaneously invasive and noninvasive measurements
*Incorrect estimate of right atrial pressure, especially with massive/torrential TR
*Suboptimal Doppler alignment (truncation, non-holosystolic signal, etc.)
*Respiratory variation
*Machine settings
*Caliper positioning (exclude fringes and transit-time linear artefacts)
*Misinterpretation of tricuspid closure artefact
*Erroneous peak velocity assignment in case of maximum velocity boundary artefact
*Theoretical limitations: neglecting the upstream velocity v_1_ in massive/torrential TR; ignoring the inertial forces; assuming complete conversion of velocity to pressure gradient

The echocardiographic estimation of pulmonary pressures traditionally relies on the SB equation, where the pressure gradient calculation is derived from the RV-to-RA CWD signal and where CVP estimates are added. However, in case of very severe TR (Grade 4 and 5), this approach may be fraught due to improper CVP estimates in these patients, as suggested by a recent analysis.^130^ In addition, the use of the SB equation to estimate the RV-to-RA gradient neglects the relatively large ‘v1’ in the unsteady Bernoulli equation (see *Figure 1*) in these conditions. Finally, the impact of the larger inertial component on the RV-to-RA gradient is also neglected. As shown in the unsteady Bernoulli equation in *Figure 1*, both ‘v1’ and the inertial term both affect the RV-to-RA gradient estimates and hence correct pulmonary pressure calculation.^131,132^

Yet, for the evaluation of pulmonary hypertension, TR velocity itself, rather than Bernoulli-derived pressure gradients or estimated absolute pressures, is recommended, in conjunction with other echocardiographic features suggestive of pulmonary hypertension, to assign a probability of its presence.^133,134^ This approach is necessary due to well-recognized pitfalls in TR velocity measurements (see *Table 4*),^135^ including the fact that small errors in velocities are squared when applying the Bernoulli equation to estimate pressure gradients, and inaccuracies in right atrial (RA) pressure estimation also often occur, particularly in severe TR.^130^ Because the right ventricle (RV) is adapted primarily for volume rather than pressure work, acute increase in afterload, such as in pulmonary embolism, typically produces systolic pulmonary artery pressures <50–60 mmHg. Higher pressures strongly suggest underlying chronic pulmonary hypertension, where RV hypertrophy has developed.^136^

**Figure 17:**
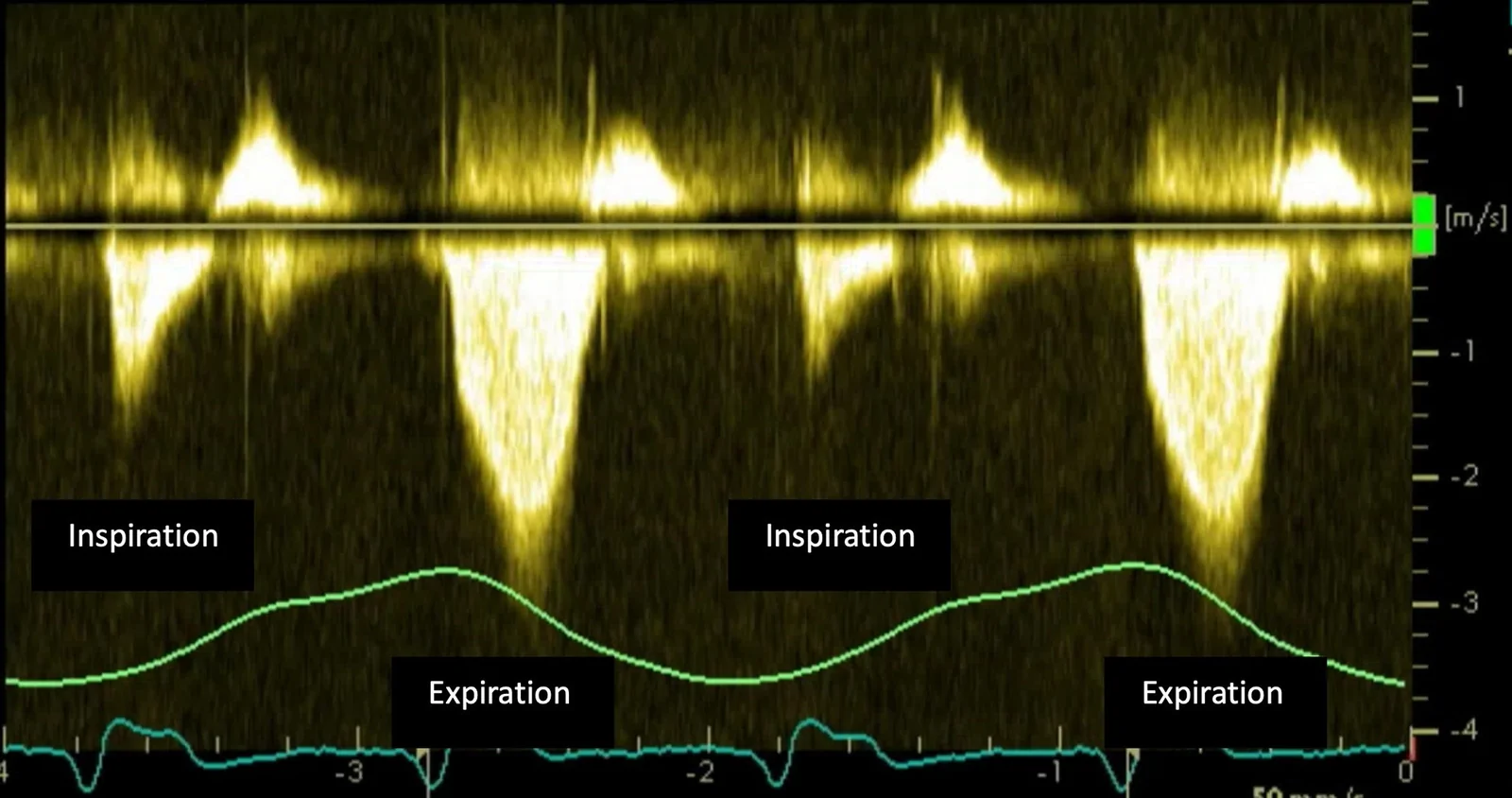
Impact of respiration on Doppler velocities in functional tricuspid regurgitation. During each inspiration, the CWD TR signal velocity profoundly decreases and adopts a v-waveform due to increased EROA during inspiration, whereas during expiration, the CWD signal becomes less triangular with higher TR velocities because EROA decreases.

Respiratory variation in TR velocities is also important to consider. During inspiration, the EROA and RegVol often increase due to annular dilation,^137,138^ making end-expiratory Doppler signals preferable for pulmonary pressure estimation (*Figure 17*). Excessive respiratory variation in TR velocities is a specific indicator of severe TR.^138^ Additional causes for discrepancies between Doppler-derived TR gradients and invasive gradients are listed in *Table 4*.^135^

Pulmonary vascular resistance (PVR) is directly related to pressure and inversely related to flow (Ohm’s law). Therefore, mean pulmonary artery pressures (mPAP) divided by cardiac output (CO) using echocardiography obtains the mPAP/CO slope, which simply represents the PVR.^139^ The mPAP/CO slope is < 3 mmHg/mL:min in healthy individuals, and this mPAP/CO slope is increasingly used during stress echocardiography to reveal abnormal pulmonary vascular disease, to check abnormal responses to increased CO, and to unveil left-sided heart disease during exercise.^140,141^ Pulmonary vascular resistance can also be estimated noninvasively by dividing peak TR velocity by the VTI at the right ventricular outflow tract (RVOT) measured by PWD. This approach has been shown to correlate with invasively measured PVR^142^ and can help distinguish pulmonary pressure elevations due to high-flow states (as may occur in anaemia, sepsis, liver cirrhosis, hyperthyroidism) from pulmonary hypertension caused by increased PVR. Several Doppler-based regression equations have been proposed to estimate mPAP or PVR,^143^ but these require meticulous Doppler assessments. While these methods demonstrate significant correlation with invasive measurements, the limits of agreement are often wide, reflecting insufficient precision for individual patient diagnosis and clinical decision-making.^144,145^

#### Continuous-wave Doppler in tricuspid regurgitation

Primary TR is uncommon, and most cases of TR are secondary or functional (FTR). A recent classification scheme in FTR divides patients into atrial functional TR (FTR), ventricular FTR (vFTR), a combination of aFTR and vFTR, and cardiac implantable electronic device-associated or device-related TR.^146^ Several observational studies have reported that FTR is independently associated with adverse outcomes across diverse population cohorts in a graded fashion.^147,148^ However, neither surgical nor percutaneous interventions for TR have yet provided definitive evidence of mortality reduction in these patients.^149–151^

Contrary to MR, peak velocities in TR can be highly variable and within a broad velocity range between 0.5 and 5 m/sec, depending on EROA, RV function, and PVR. The extent of the colour Doppler TR jet in the right atrium (RA) is most dependent on the momentum flux (M), which has a squared relation with TR velocity and is described as M = EROA × TR velocity.^2,95^ Because previous *in vitro* experiments described the relation between colour Doppler jet area as $\mathrm{Jet}\;\mathrm{Area}=1.25\;x\;M^{0.28}$,^95^ this implicates that for the same RegVol, an increase in TR velocity of 2.5 m/sec to 5 m/sec will increase the colour Doppler jet area in the RA with 30%, thus not fourfold. However, because colour Doppler jet area is affected by many variables including instrument settings, guidelines do not recommend to assess the severity of atrioventricular regurgitation based on colour Doppler jet area.

**Figure 18:**
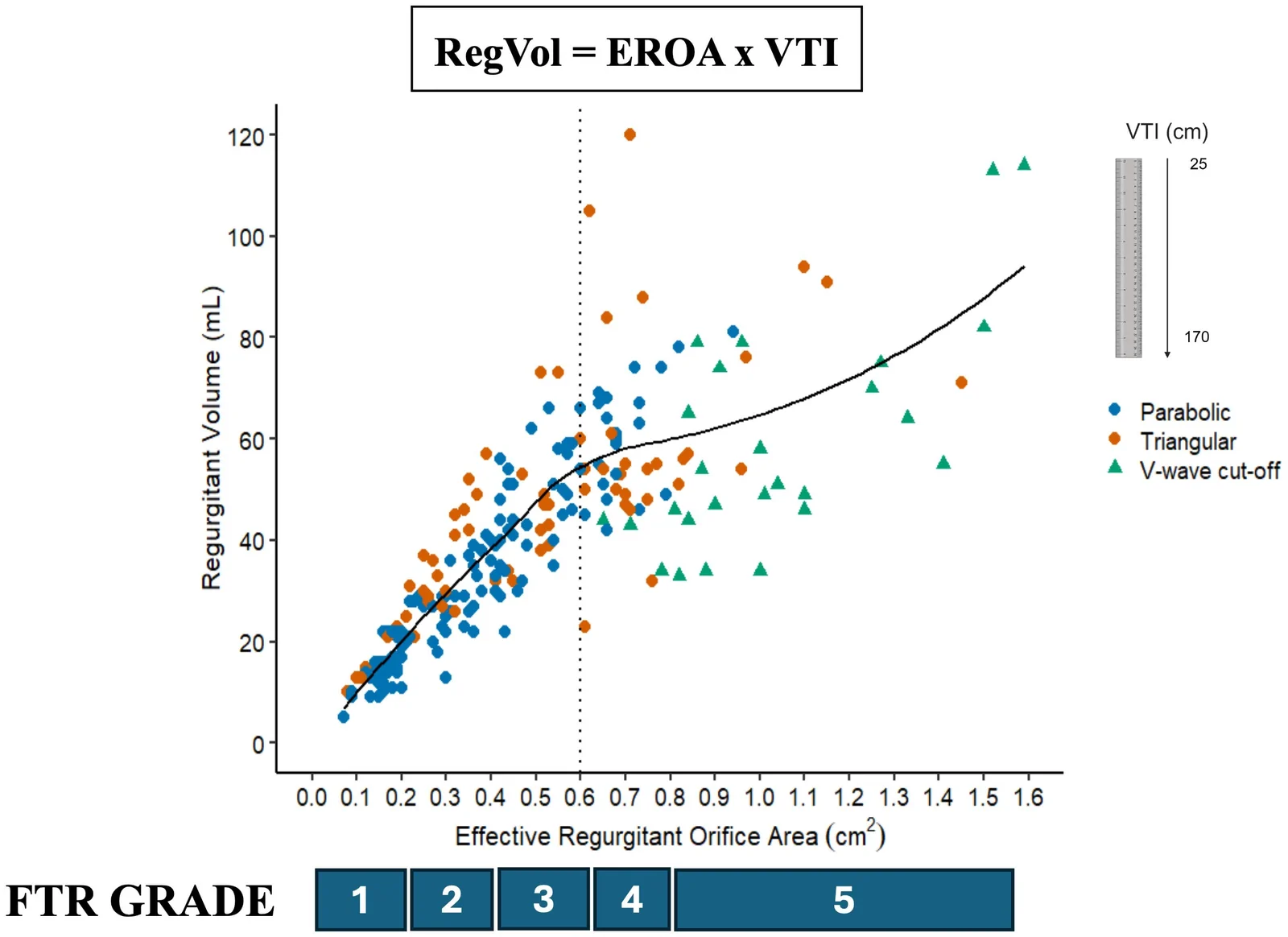
Relationship between effective regurgitant orifice area and regurgitant volume in functional tricuspid regurgitation. The VTI is the slope linking EROA to RegVol. This relation is initially linear (black line), but at higher EROAs > 0.6 cm2 (threshold indicated by the dotted line), a non-linear relation develops with attenuated VTI and eventually less increase of RegVol in FTR TR Grade 4 and 5. This is especially the case for patients with v-wave cut-off sign (green triangles). Modified figure from Reference 4, with permission.

As mentioned above, the relationship between EROA and RegVol is determined by the VTI (the RgVol/EROA slope), and this relationship in general is linear for both FMR and FTR. However, attenuation of the VTI occurs at very high EROAs, particularly in very severe FTR, i.e. TR Grade 4 or 5 (see *Figure 18*).^4,109^ Consequently, although EROA increases along the TR classification from Grade 1 to 5, the increase of RegVol is attenuated from Grade 4 towards Grade 5. Therefore, TR RegVol may ‘underestimate’ the severity relative to the EROA in cases of massive or torrential TR.^4^

#### The shape of the tricuspid regurgitation continuous-wave Doppler signal

**Figure 19:**
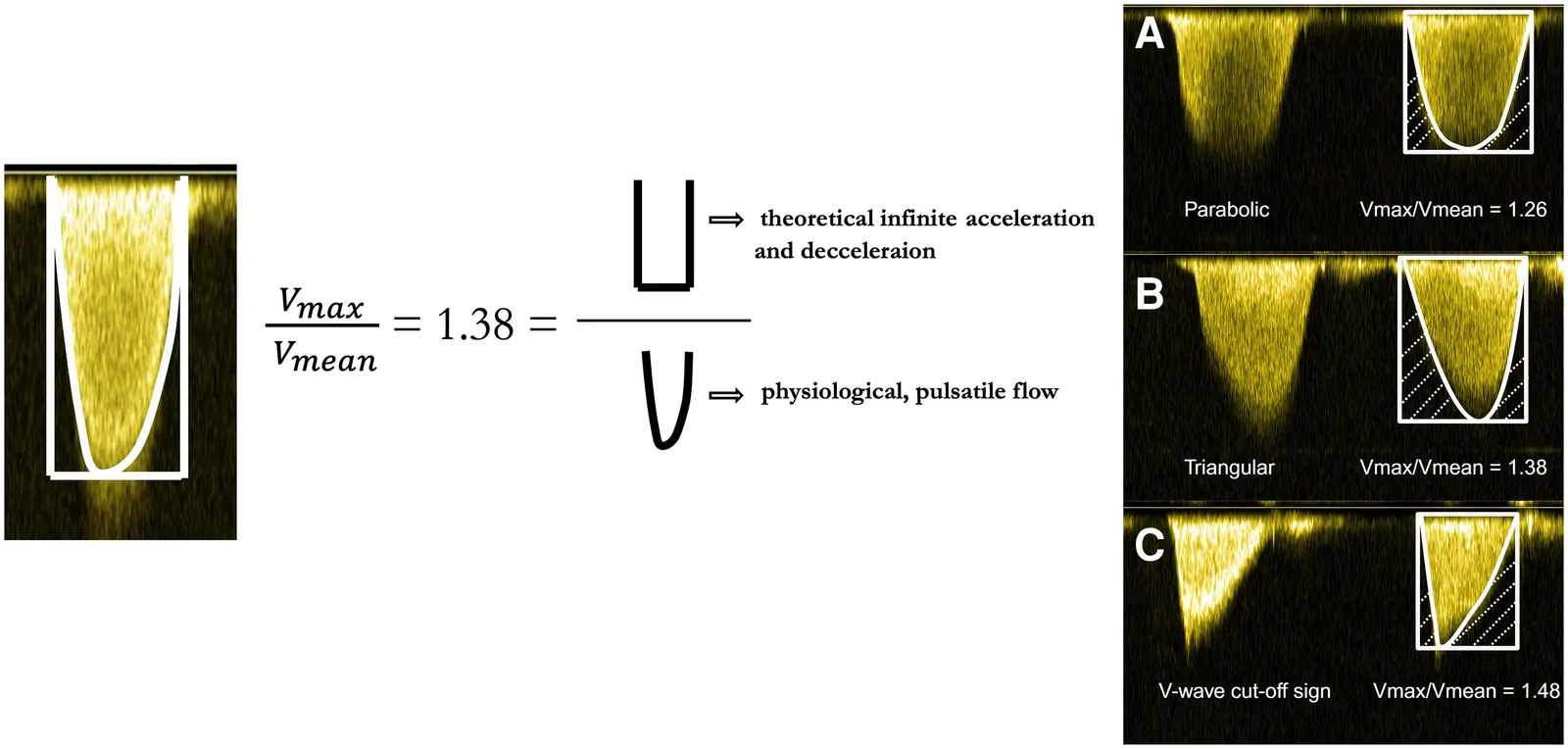
The continuous-wave Doppler shape spectrum in functional tricuspid regurgitation. Vmax/Vmean ratio of the CWD signal represents the quotient of the theoretical rectangular area and the area occupied by the CWD signal, in this case Vmax/Vmean ratio of 1.38, meaning that the rectangular area is 38% larger than the CWD Doppler area. This is also equivalent to the ratio of the VTI of the rectangle and the VTI of the CWD signal. Right panel: in most patients with mild, moderate, and severe FTR (FTR Grade 1, 2, and 3), the CWD signal has a parabolic shape (*A*), whereas in massive FTR (Grade 4), the shape is mostly triangular (*B*). In many patients with torrential FTR (Grade 5), the signal is denoted as v-wave cut-off signal (*C*). The degree of ‘triangularity’ can be represented by Vmax/Vmean value of the Doppler signal, which corresponds to the shaded surface area between the imaginary rectangle and the area covered by the Doppler signal. The Vmax/Vmean value in FTR represents an integrative hydraulic severity measure.

Similar to MR, the shape of the CWD envelope in TR is fundamentally determined by the time-varying transvalvular pressure gradients, which itself arise from the complex and dynamic interplay between flow and the corresponding impedance, consistent with Ohm’s law.^4^ In contrast to FMR, FTR exhibits a spectrum of CWD shapes ranging from parabolic to more triangular shapes, with the v-wave cut-off at the extreme of the ‘triangularity’ spectrum (*Figure 19*).^4^ Beyond the flow and impedance as determinants of the CWD shape, factors such as Doppler beam-to-jet angulation, acute pressure changes, and cardiac rhythm disturbances may also alter the Doppler envelope morphology.

The v-wave cut-off phenomenon, often observed in torrential FTR,^4^ occurs when the highest instantaneous RV-to-RA pressure gradient develops early due to low atrial impedance, followed by progressive gradient reduction until near equalization of ventricular and atrial pressures.^4^ In these cases, peak RV pressure occurs later than the peak transvalvular gradient, whereas in non-v-wave cut-off signals, peak ventricular pressure coincides with the maximal transvalvular gradient.^132^ Patients with a v-wave cut-off pattern typically have markedly elevated RA pressures, low transvalvular Doppler gradients (‘ventricularization’ of atrial pressures), and poor clinical outcomes.^26^ Importantly, the presence of the v-wave cut-off sign may theoretically not allow accurate estimates of pulmonary pressures when using the SB equation, i.e. the RV–RA gradient estimates may be flawed due to (i) the increased inertial term in the unsteady Bernoulli equation, and (ii) the use of the SB in this condition neglects the v_1_ velocities (*Figure 1*).^131,132^ However, improper pulmonary pressure calculations in very severe TR are largely attributed to suboptimal estimates of central venous pressure due to classification constraints, rather than improper RV-to-RA gradient assessments.^4,26,130^ Therefore, in patients with very severe TR (Grade 4 and 5) with unreliable central venous pressure estimates, invasive pressure assessments are recommended, specifically when considering surgical or percutaneous TR intervention.

The CWD signal morphology has been incorporated into severity assessment algorithms of atrioventricular regurgitation.^78,152^ A recent study proposed the echocardiographic peak-to-mean velocity ratio as measure of triangularity of the CWD signal in FTR, demonstrating predictive value for clinical outcomes (see *Figure 19*).^4^ The Vmax/Vmean ratio of the TR CWD signal was also shown to provide prognostic value in patients treated for precapillary pulmonary hypertension, but the mechanism and waveform driving the CWD ‘triangularity’ differs from the CWD waveform observed in significant FTR.^153^ Because the Vmax/Vmean triangularity index is affected by pulmonary pressures, RV function, and EROA, it can be considered an integrative hydraulic severity measure of the complex RV–PVR–RA–PA conundrum, rather than a surrogate for any of these components,^4^ and its broader use needs further validation. Finally, TR duration corrected for systolic time (TRDc) is also associated with TR severity as well as RV function, and it is associated with adverse outcomes in these patients.^137,154^

### Tricuspid stenosis

Tricuspid stenosis (TS) may occur in native valves following rheumatic disease, carcinoid heart disease, or congenital disease. Continuous-wave Doppler allows to assess mean inflow gradients, whereas PHT-based calculation (220/T1/2, as originally derived for MS) of the tricuspid valve area is less accurate, with some data suggesting the use of an alternative constant (190/T1/2).^155^ The latter equation has not been well validated, and routine clinical use is not encouraged.

The continuity equation is an alternative approach for the assessment of valve area in TS, in the absence of significant TR. Finally, TS area can be calculated as ‘stroke volume/tricuspid valve VTI’, with stroke volume measured from either the RV or LV outflow. This method is however time-consuming, it cannot be applied in the presence of significant regurgitation from another valve, and it requires further validation.^156^

## The pulmonary valve

### Pulmonary regurgitation

**Figure 20:**
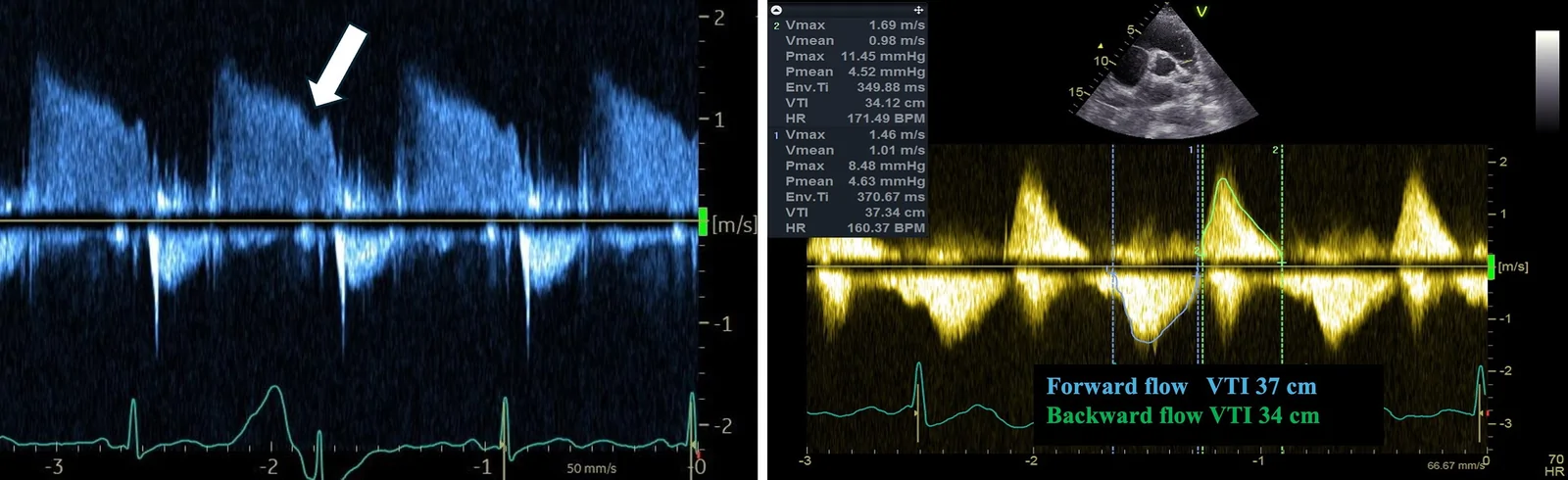
Continuous-wave Doppler signal in pulmonary regurgitation. Left panel shows a typical CWD signal in a patient with mild PR, with high early velocity following valve, a slow deceleration due to decreasing PA–RV gradient during diastole until the lower end-diastolic velocity is reached with fast pressure gradient drop towards pressure equalization. The white arrow indicates the A-dip, i.e. a fast decline in velocity due to atrial kick. Note also that in mild PR, the VTI of the PR signal is much larger than the forward signal. In the right panel, a severe PR CWD signal is shown. The CWD signal in severe PR is smaller and intense (almost equal pixel intensity compared to the forward signal) and with a fast deceleration slope, resulting in a small backward VTI of 34 cm (green tracing), almost similar to the forward VTI (blue tracing), indicating very large EROA.

**Figure 21:**
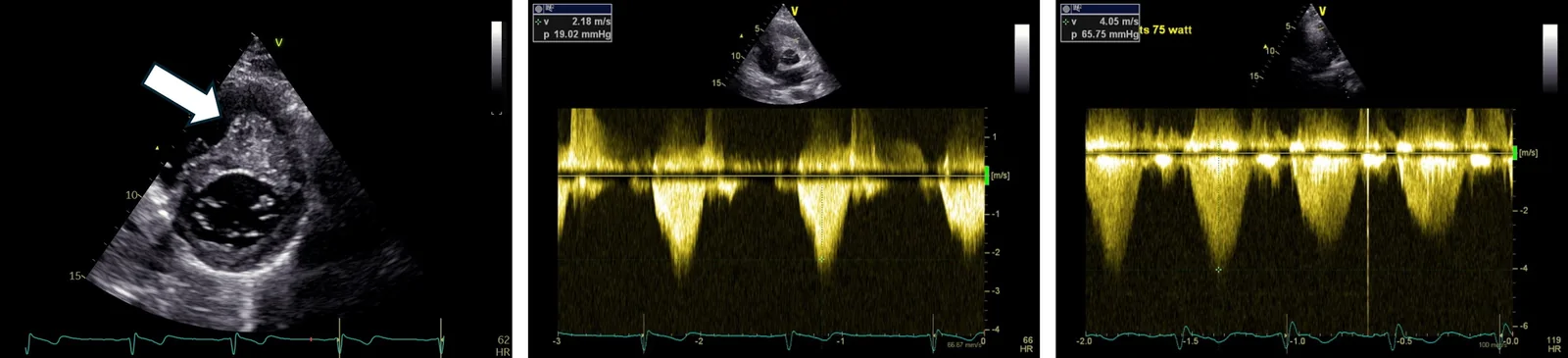
Continuous-wave Doppler signal in right ventricular outflow tract obstruction. Left panel shows a hypertrophic right supraventricular crest of the right ventricle (arrow) with an RVOT gradient of 19 mmHg at rest (central panel), increasing to an RVOT obstruction peak gradient of 66 mmHg with typical dynamic dagger-shaped CWD signal during bicycle stress echocardiography (right panel).

Trace to mild pulmonary regurgitation (PR) is a common echocardiographic finding and may be present in healthy individuals.^157,158^ Primary PR is most often related to congenital abnormalities or pulmonary hypertension, whereas moderate to severe PR is frequently encountered in patients following surgical repair of the tetralogy of Fallot. In mild to moderate PR, CWD profile is characterized by a rapid rise in flow velocity immediately after pulmonary valve closure. After reaching peak early diastolic velocity, the signal demonstrates a gradual deceleration governed by the progressive decline in the pulmonary artery–RV diastolic pressure gradient until pulmonary valve opening, followed by rapid cessation (*Figure 21*).^159,160^ In contrast, severe PR is associated with a more triangular CWD envelope, reduced VTI, and a steep deceleration slope, resulting in a shortened PHT (*Figure 21*). A PHT <100 msec or a deceleration time <260 msec has been shown to be consistent with severe PR.^78^ Additionally, in severe PR, the regurgitant signal intensity often approaches that of the antegrade systolic flow (*Figure 20*), and premature termination of diastolic regurgitant flow may be observed. As in AR, the CWD profile of PR is not determined solely by the regurgitant orifice area but is also influenced by RV myocardial properties. Increased RV stiffness and elevated filling pressures may result in accelerated deceleration slopes and early termination of PR flow, even in the absence of a very large regurgitant orifice.

Pulmonary artery pressures can be estimated using PR velocities.^143^ These methods allow estimation of mean and diastolic pulmonary artery pressures. However, several important limitations must be considered. Accurate alignment of the Doppler interrogation with the PR jet is essential, as suboptimal alignment may lead to underestimation of regurgitant velocities. Inspection of the antegrade flow envelope complemented by PWD interrogation of the RVOT, including assessment of AT, notching, and the presence of diastolic forward flow, can further enhance the assessment of pulmonary circulation haemodynamics. Moreover, restrictive RV physiology or pericardial constriction can substantially alter the PR Doppler profile and reduce the reliability of pressure estimation. Specifically, constrictive physiology typically produces high early diastolic PR velocities followed by abrupt deceleration and low end-diastolic velocities. Finally, in severe PR, as mentioned above, rapid deceleration and low end-diastolic velocities preclude reliable estimation of diastolic pulmonary artery pressure using this approach.

### Pulmonary stenosis

In PS, careful visual inspection is essential to distinguish intrinsic valvular disease from supravalvular narrowing or RVOT obstruction. The latter may be encountered in fixed obstruction or with prominent muscle bands, such as double-chambered RV or obstructive hypertrophic cardiomyopathy (*Figure 21*). Pulsed wave Doppler analysis can aid in localizing the level of obstruction by systematically repositioning the sample volume along the RVOT, across the pulmonary valve, and into the supravalvular region.

## Future perspective: the Doppler signal as a masterclass for artificial intelligence

Specific Doppler velocity profiles, their timings and peaks, specific slopes, shapes, and signal spectral intensities often coexist.

Artificial intelligence systems are uniquely positioned to assimilate this rich, high-density information. Rather than relying on isolated metrics, AI can simultaneously integrate multiple Doppler attributes—both those readily recognizable to human observers and additional latent features that are imperceptible or impractical to quantify manually. Through pattern recognition across large datasets, AI models may identify subtle combinations of waveform morphology, timing offsets, beat-to-beat variability, and spectral texture that collectively encode diagnostic and prognostic information.

In valvular heart disease, such an approach has the potential to move Doppler interpretation beyond single-parameter thresholds towards a holistic, physiology-driven assessment. By learning from comprehensive Doppler signatures across diverse clinical states, AI could support clinicians in differentiating disease severity, identifying discordant findings, recognizing complex or mixed valvular lesions, and contextualizing Doppler measurements within the broader haemodynamic environment. Importantly, this AI-augmented interpretation should be viewed not as a replacement for expert judgement, but as a complementary tool —enhancing consistency, reducing cognitive load, and uncovering clinically meaningful patterns that extend beyond traditional human-centric analysis.^117^

## Take home messages

1 Optimize acquisition before interpretation: Accurate Doppler assessment hinges on proper beam alignment, multiple acoustic windows, and careful adjustment of gain and wall filters; technical errors and potential echo artefacts remain a major cause of misinterpretation.

2 Interpret Doppler signals in the right physiological context: Velocities and gradients are flow-dependent; always integrate Doppler findings with flow state, loading conditions, and complementary parameters.

3 Use waveform morphology as a diagnostic tool: The shape, timings, and intensity of the Doppler envelope provide key insights into lesion severity and underlying haemodynamics, often beyond velocity values alone.

4 Adopt a multiparametric and multimodality approach in complex cases: Discordant findings, mixed valve disease, or altered flow states require integration of multiple Doppler indices and, when needed, adjunctive imaging using CT or CMR or invasive assessment.

5 Maintain critical oversight despite automation: Automated and AI-assisted measurements can enhance efficiency, but careful visual validation of Doppler signals remains essential to avoid systematic errors.

## Conclusion

Spectral Doppler echocardiography remains a cornerstone in the comprehensive evaluation of valvular heart disease. Its underlying principles, grounded in fluid dynamics and the conservation of energy, offer profound insights into intracardiac haemodynamics that extend well beyond simple velocity measurements. Accurate application, however, requires meticulous attention to signal acquisition, careful interpretation of waveform morphology and timing, and thoughtful integration with complementary echocardiographic parameters and multimodality imaging. Although the principles outlined in this review are relatively straightforward, real-world cases often involve complex haemodynamic derangements. For example, intracardiac shunts can alter transvalvular flow and pressure relationships, thereby confounding Doppler-based assessment of valve lesions; such effects must be carefully considered in comprehensive haemodynamic evaluation.

In an era increasingly dominated by advanced structural imaging, there is a compelling need to revive and re-emphasize the role of Doppler echocardiography as a bedside physiological tool. When applied at the point of care, Doppler interrogation allows real-time haemodynamic assessment, immediate correlation with clinical findings, and dynamic evaluation under changing loading conditions—capabilities that are particularly valuable in unstable patients and complex valvular syndromes. Re-engaging clinicians with the fundamental interpretation of Doppler signals fosters deeper physiological understanding, enhances diagnostic confidence, and reduces overreliance on isolated numerical outputs.

As imaging technology and analytic methodologies continue to evolve, a critical and nuanced approach to Doppler echocardiography rooted in bedside practice and physiological reasoning will remain essential for precise diagnosis, meaningful risk stratification, and informed therapeutic decision-making in patients with valvular heart disease. Rather than being superseded by innovation, Doppler echocardiography should be revived as the foundation upon which advanced imaging tools are built and interpreted. Current available AI tools in echocardiography are largely focused on automated measurements and detection of predefined features, often replicating human workflows with improved efficiency and reproducibility. In contrast, future AI applications are expected to move beyond task automation towards integrative, physiology-driven interpretation, i.e. by synthesizing the full Doppler signal, capturing complex temporal, morphological, and spectral Doppler patterns to provide deeper diagnostic and prognostic insights in valvular heart disease.

## Data Availability

No AI tools were used for this manuscript.
