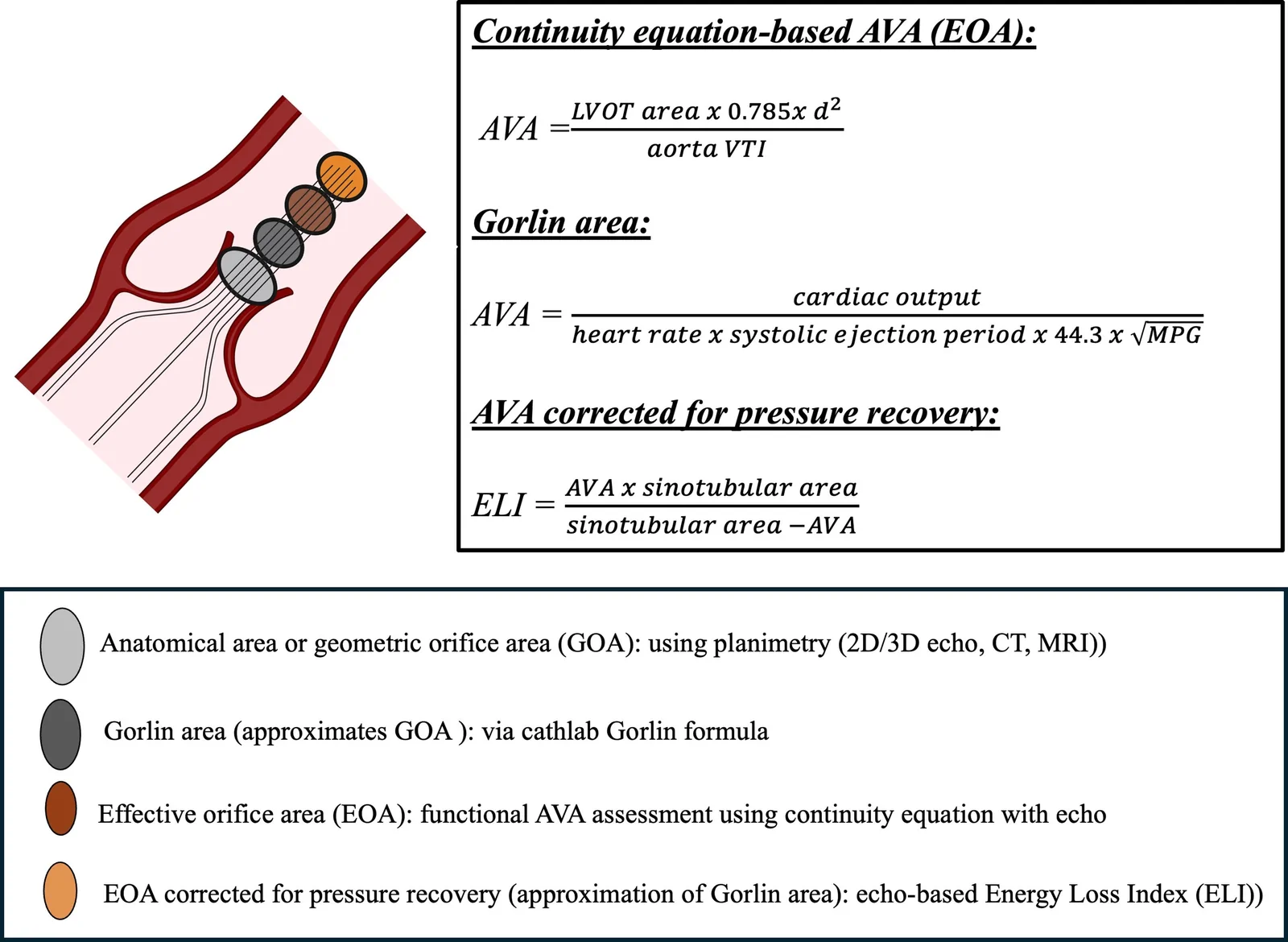# Correction to: Continuous-wave Doppler interrogation in valvular heart disease: pearls and pitfalls

**DOI:** 10.1093/ehjimp/qyag180

**Published:** 2026-11-13

This is a correction to: Frank Timmermans, Edgar Argulian, Hatem Soliman-Aboumarie, Continuous-wave Doppler interrogation in valvular heart disease: pearls and pitfalls, *European Heart Journal - Imaging Methods and Practice*, Volume 4, Issue 1, January 2026, qyag096, https://doi.org/10.1093/ehjimp/qyag096

In the originally published version of this manuscript, the caption for Figure 4 erroneously referred to a “[…] highly calcified bicuspid aortic valve with a Δ*p* max. of 30.7 mmHg obtained in the apical chamber view […]”.

This has been corrected to read “[…] highly calcified bicuspid aortic valve with a Δ*p* max. of 52 mmHg obtained in the apical chamber view […]”.

Additionally, an error in the Continuity equation-based AVA (EOA) within Figure 6 has been corrected.

The corrected Figure 6 reads:

**Figure:**
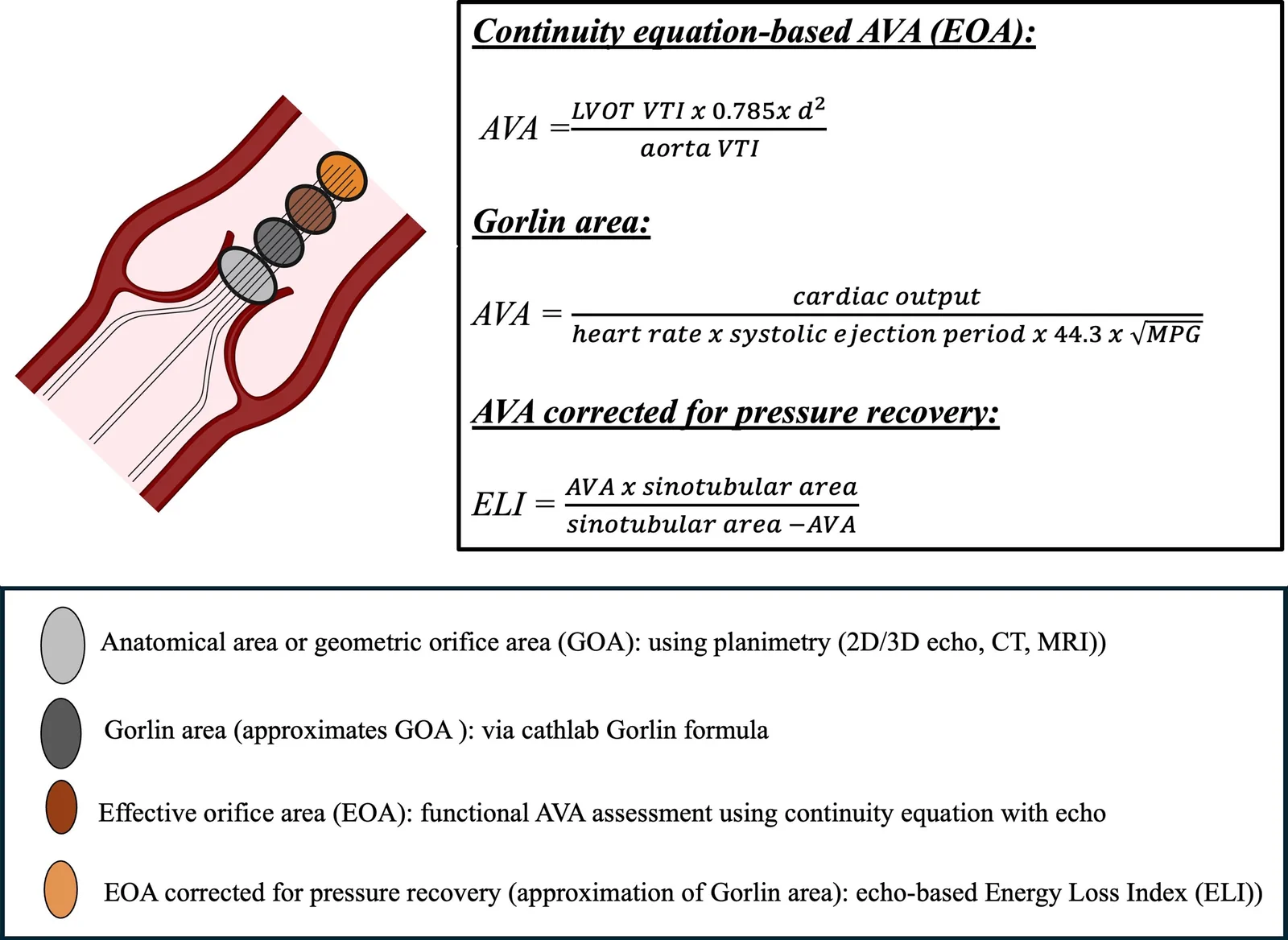


Instead of:

**Figure:**